# Fecal microbiota transplantation: application scenarios, efficacy prediction, and factors impacting donor-recipient interplay

**DOI:** 10.3389/fmicb.2025.1556827

**Published:** 2025-03-25

**Authors:** Yaxin Liu, Xinru Li, Yuchao Chen, Qinyan Yao, Jinjie Zhou, Xiaoxuan Wang, Qingguo Meng, Jiaxuan Ji, Zihan Yu, Xin Chen

**Affiliations:** ^1^Department of Gastroenterology and Hepatology, Tianjin Medical University General Hospital, Tianjin, China; ^2^Tianjin Institute of Digestive Disease, Tianjin Medical University General Hospital, Tianjin, China

**Keywords:** gut microbiota, fecal microbiota transplantation, donor-recipient interplay, prediction model, safety and efficiency

## Abstract

Fecal microbiota transplantation (FMT) represents a therapeutic approach that directly regulates the gut microbiota of recipients, normalizes its composition and reaping therapeutic rewards. Currently, in addition to its general application in treating *Clostridium difficile* (*C. difficile*) infection (CDI), FMT treatment has also been extended to the fields of other gastrointestinal diseases, infections, gut-liver or gut-brain axis disorders, metabolic diseases and cancer, etc. Prior to FMT, rigorous donor screening is essential to reduce the occurrence of adverse events. In addition, it is imperative to evaluate whether the recipient can safely and effectively undergo FMT treatment. However, the efficacy of FMT is influenced by the complex interactions between the gut microbiota of donor and recipient, the degree of donor microbiota engraftment is not necessarily positively related with the success rate of FMT. Furthermore, an increasing number of novel factors affecting FMT outcomes are being identified in recent clinical trials and animal experiments, broadening our understanding of FMT treatment. This article provides a comprehensive review of the application scenarios of FMT, the factors influencing the safety and efficacy of FMT from the aspects of both the donors and the recipients, and summarizes how these emerging novel regulatory factors can be combined to predict the clinical outcomes of patients undergoing FMT.

## 1 Introduction

The gut microbiota is composed of trillions of different microorganisms, bacteria, archaea, phages, and protozoa, which forms a solid organ weighing ~2 kg (Ruan et al., 2020). The gut microbiome contributes to the homeostasis in human, which is responsible for nutrient digestion, metabolism regulation, resistance to external pathogens, and modulation of host immune responses (Ghani et al., 2024). The value of live biotherapeutic products, which act directly in the gastrointestinal tract and alter microbial community of the recipient, has been identified in the treatment of microbiome disruption-related diseases (Goldsmith et al., 2024). Fecal microbiota transplantation (FMT) is a therapy performed by oral, enteral, or colonic administration of donor feces containing natural microbiota, restoring the disrupted microbial community into a healthy one (Gupta and Khanna, 2017). At present, FMT has been recognized as an innovative therapeutic approach from the two main aspects: its curative effect in the treatment of *Clostridioides difficile* infection (CDI) and the expanded comprehension of the intricate interplay between the gut microbiota and human wellbeing (Afzaal et al., 2022). In addition to the delightful treatment outcomes observed in CDI, FMT has also been investigated for a wide range of diseases related to dysbiosis, including inflammatory bowel disease (IBD) (Costello et al., 2017), irritable bowel syndrome (IBS) (Johnsen et al., 2018), metabolic syndrome (MetS) (Proença et al., 2020), neuropsychiatric diseases (Settanni et al., 2021) and autoimmune diseases (Huang et al., 2022), etc. As the interest in FMT continues to surge, a great number of clinical studies are currently employing FMT for the treatment of more than 80 diseases, showing promising safety and effectiveness (Wang Y. et al., 2022; Karimi et al., 2024).

Nevertheless, variations in the therapeutic efficacy of FMT are observed when treating different patients with the same disease, suggesting that a multitude of factors influence the effectiveness of FMT. In terms of the donor, it involves donor screening, baseline characteristics of the donor, such as health condition (presence or absence of underlying diseases), lifestyle, body weight, gender, age, genetic relatedness, etc, as well as the microorganism constituents within the donor's gut, including the microbiota, virus and fungal. Several recent guidelines have been published to direct the treatment process for FMT (Cammarota et al., 2017; Lopetuso et al., 2023), however, part of the restrictions for donors are not mandatory, and several factors are not explicitly stated in current guidelines, suggesting that we should consider the importance of these factors in FMT from multiple perspectives. Regarding the recipients, firstly, the indication of FMT should be considered. Besides the extended diseases mentioned above, which have the potential to be treated by FMT, other baseline status of the recipients are required to be considered, for example, the immune system function of the recipient, to reduce the occurrence of adverse events (Shogbesan et al., 2018). Furthermore, from the aspect of efficiency of FMT, factors such as bowel preparation, drug usage, diet and lifestyle, etc, are taken into account. The severity of the patient's disease also affects the efficacy of FMT, more severe or recurrent conditions may require repeated FMT procedures or be exhibit no response to FMT.

It is worth noting that the interplay between the donors and the recipients is a dominant factor of FMT, defined as “donor-recipient compatibility”. For example, the potential impact of sex-discordant FMT and age-discrepant FMT warrants more attention (Benítez-Páez et al., 2022; Sehgal et al., 2024). More importantly, mechanistically, as a therapy based on gut microbiota, the gut microorganisms from the donor are introduced into the gut of recipients after FMT. With the invasion of exogenous microorganisms, the baseline microbial community is disturbed, subsequent alteration of gut microbiota occurs within recipient's gut, and ultimately, a newly constituted equilibrium state of microbiota is reached. Generally, the engraftment of donor-derived microorganisms is the mostly considered physiological process post-FMT, however, the succession of gut microbiota following FMT extends far beyond the mere microbial engraftment. Various possible ecological scenarios within the gut of recipients could be observed, which depends on the baseline gut microbiota of both colonizers and residents, further emphasizing the importance of donor-recipient interplay (Schmidt et al., 2022; Wilson et al., 2021). Moreover, studies also showed that engraftment of microbial components from the donor may not be necessarily correlated with clinical improvement (Browne et al., 2021). It is of significant importance to clarify the patterns of post-FMT microbial changes within the recipient's gut, and how the ultimate microbial community and the subsequent long-term physiological changes affect the prognosis of diseases.

To sum up, the success of FMT therapy is determined by a complex regulatory network involving multiple factors. Thus, the therapeutic efficacy-related factors mentioned above can be extended to two application scenarios: (1) predicting the outcomes of FMTs that have been conducted; {2) selecting the optimal donors according to donor-recipient matching scheme prior to FMT. Recently, however, more and more researchers are exploring the treatment effect of FMT after carefully donor screening, while several studies resulted in contradictory conclusions (Caenepeel et al., 2024; Zhang Y. et al., 2024). This confusing phenomenon reminds us that still numerous unknown factors involved in this complex regulatory process, acting as promotors and inhibitors in the FMT process. There is still a considerable journey ahead to elucidate the optimal regimen for FMT therapy.

Innovatively, recent animal experiments are contributing to revealing potential participants in the FMT process, which could be partially explained by the difference of microbiota, and the results of these studies are gradually being extended to randomized controlled trials for validation. Furthermore, researchers are committing to the development predictive models for the efficacy of FMT (He et al., 2022), which integrate a variety of variables, including general characteristics and microbiome features of individuals, etc., which contributes to further refining more standardized procedures of FMT. In the future, the illumination of regulatory mechanisms will also be conducive to enhance the explainablity and transparency of predictive models.

In our review, we first summarized the current therapeutic status of FMT in various diseases, the microbial alterations in these FMT-targeting diseases and potential microorganism-related mechanisms. Moreover, our review systematically integrated the latest results of clinical researches and animal experiments in the field of FMT, which aims to summarize the currently recognized factors which should be paid considerable attention in the process of FMT, emphazing the factors which have always been overlooked, and discussing the values of newly discovered factors affecting the success rate of FMT.

## 2 Current applications of FMT

Many diseases are characterized by functional changes in composition and gut microbiota. When gut homeostasis is disrupted, normal body functions are impaired and gastrointestinal and extra-gastrointestinal disorders arise, including recurrent CDI (rCDI), IBD, metabolic disorders, cancer, etc. Dysbiosis of 479 gut microorganisms has been reported to be associated with 117 gastrointestinal and extra-gastrointestinal diseases (Zhang X. et al., 2023). Microbial dysbiosis-related diseases have the potential to be alleviated via FMT.

The safety and efficacy of FMT in rCDI have been extensively evaluated. In a systematic review that included 317 patients treated in 27 case series and reports, 92% of the individuals showed disease remission (Gough et al., 2011). Early FMT improves survival in severe CDI. A retrospective cohort study described 111 patients with severe CDI, including 66 patients treated with FMT and 45 patients who did not receive FMT, and the three-month mortality rate after diagnosis of severe CDI in the FMT-treated patients was 12% (8/66), compared with 42% (19/45) in the standard-treatment group, *p* < 0.001 (Hocquart et al., 2018). Six clinical studies, enrolling 320 subjects, found that the use of FMT in immunocompetent rCDI participants may lead to a substantial increase in the remission of rCDI in the FMT group compared to the control group (Minkoff et al., 2023). FMT also appears to be beneficial for CDI subtypes. For severe or fulminant CDI, the overall successful remission rate for a single FMT was 0.88 (Song et al., 2022). Notably, however, severe CDI, severe-complicated indication, number of previous CDI-related hospitalizations and inpatient status are independent predictors of failure in single FMT in patients with rCDI (Beran et al., 2023; Fischer et al., 2016; Ianiro et al., 2017). Level of fecal calprotectin concentration of CDI patients before first FMT procedure is associated with the need of repeat FMT (Gallo et al., 2020).

Crohn's disease (CD) and ulcerative colitis (UC) are defined as IBD, which are chronic inflammatory diseases that affect parts of the gastrointestinal tract and may even manifest with additional intestinal symptoms. A growing body of evidence supports the role of gut microbiota in the pathogenesis of IBD. Pooled results from four RCTs showed FMT to be superior to placebo in active UC, with the endpoint defined as endoscopic remission (Costello et al., 2017), which is also supported by several other researches (Paramsothy et al., 2017; Tang et al., 2020). In an animal study, FMT from healthy human donor reduced the susceptibility to colitis in dextran sulfate sodium (DSS)-induced germ-free mice, while UC bacteria promoted the expression of inflammatory markers and pro-inflammatory factors (Yang et al., 2022). According to the international Rome consensus conference on gut microbiota and FMT in IBD, FMT may be effective in the induction of remission for mild to moderate UC, while there is insufficient evidence to recommend FMT as a treatment for UC in routine clinical practice (Lopetuso et al., 2023). Investigations for pouchitis and CD are ongoing (Lee and Chang, 2021). However, there is insufficient evidence on the safety and efficiency to recommend FMT as a treatment for CD and pouchitis in clinical practice (Lopetuso et al., 2023). In short, FMT is a promising alternative or adjunct to current therapies for patients with UC and CD. More extensive clinical trials are pending to confirm its long-term efficacy and safety.

The results of current studies vary on the efficacy of FMT in IBS. In a RCT examining the effects of FMT delivered via colonoscopy in patients with IBS, the IBS severity scores 3 months after single donor FMT was lower than that after autologous FMT, indicating the effectiveness of FMT on IBS (Johnsen et al., 2018). Other studies have shown that FMT can effectively improve the abdominal symptoms, fatigue and quality of life of IBS patients. For example, the response rates of FMT groups after 3 months were significantly higher than that of the placebo group, and the improvement of symptoms was correlated with the change of intestinal microbiota, which further confirmed the positive effect of FMT on IBS (El-Salhy et al., 2020). However, in a randomized, double-blind, placebo-controlled trial that included patients with moderate to severe IBS, a significant difference in improvement in IBS-severity scoring system (IBS-SSS) scores was observed 3 months after treatment in favor of placebo, but not FMT (Halkjær et al., 2018). Additionally, in another clinical trial, two FMTs 4 weeks apart did not significantly reduce IBS-SSS scores, although the improment of abdominal distension was more observed in patients receiving FMT and was associated with a reduction in hydrogen sulfide-producing bacteria (Yau et al., 2023). These findings suggests that changes in the gut microbiota may not be sufficient to fully achieve clinical improvement of IBS, and further in-depth studies on the mechanism of action of FMT and optimization of treatment regimens are needed to improve its therapeutic efficacy in IBS.

Generally, studies pertaining to FMT has predominantly focused on the aforementioned gastrointestinal diseases. Overall, the AGA Clinical Practice Guideline on FMT for select gastrointestinal diseases provided recommendations on the use of FMT in adults with rCDI, severe to fulminant CDI, IBD (including pouchitis) and IBS, adhering to the perspective that FMT is recommended in CDI under certain conditions, while this therapy cannot yet be recommended in other gastrointestinal conditions (Peery et al., 2024).

FMT also has potential applications in other diseases. More than 200 clinical trials have been conducted on the use of FMT in the treatment of various inflammatory and autoimmune diseases and cancer. FMT has emerged as a potential treatment for severe colitis associated with graft-vs.-host disease (GvHD) after hematopoietic stem cell transplantation (Zhang et al., 2021). In cancer, studies have shown that FMT improves immune checkpoint inhibitor (ICI)-associated colitis in cancer patients, accompanied by reconstitution of the intestinal flora and a relative increase in the proportion of regulatory T cells in the colonic mucosa (Wang et al., 2018). In a non-randomized clinical trial, after FMT in two enemas one week apart, five individuals subjectively reported improvement in immune-mediated dry eye 3 months after FMT (Watane et al., 2022). Germ-free mice transplanted with microbiota from MS patients exhibited a reduced proportion of IL-10 Tregs compared to mice with microbiota from healthy controls. Further investigation of immunomodulatory mechanisms suggests that multiple sclerosis (MS)-associated bacterial species reduce Tregs and increase Th1 lymphocyte differentiation *in vitro*, while exacerbating disease severity (Berer et al., 2017). To date, participants with psoriatic arthritis (PsA) have found FMT to be acceptable and safe. A double-blind, randomized, placebo-controlled trial (NCT03058900) is underway to determine whether FMT is more effective than placebo in reducing disease activity in patients with PsA and active peripheral arthritis treated with weekly subcutaneous methotrexate injections (Kragsnaes et al., 2021). A study in mice determined that FMT alleviates the severity of lupus by repairing antibiotic-induced dysbiosis in the gut flora, suggesting that manipulation of the gut microbiota is a logical and promising novel therapeutic strategy for systemic lupus erythematosus (SLE) (Zhang et al., 2020). The first clinical trial of FMT in patients with active SLE (ChiCTR2000036352) suggested that FMT may be a feasible, safe, and potentially effective short-term treatment for patients with SLE, which effectively shifted the gut microbiota to anti-inflammatory and improved clinical parameters (Huang et al., 2022). In type 1 diabetes (T1DM), a RCT in Netherlands showed that FMT could stabilized residual β-cell function of patients (de Groot et al., 2021).

Moreover, the value of FMT in metabolic dysfunction-associated fatty liver disease (Abenavoli et al., 2024), spesis (Keskey et al., 2020), anti-aging, neuropsychiatric disorders (Bruggeman et al., 2024), obesity and metabolic syndrome (Zhang Z. et al., 2024), etc, are also being explored in both human cohort trails and animal experiments. A systematic literature review including 782 studies investigated the clinical FMT uses in 85 specific diseases, showing the promising future of FMT for dysbiosis-related diseases within and beyond the gut (Wang Y. et al., 2022).

## 3 The fundamental patterns of changes in the gut microbiota post-FMT

**Figure 1:**
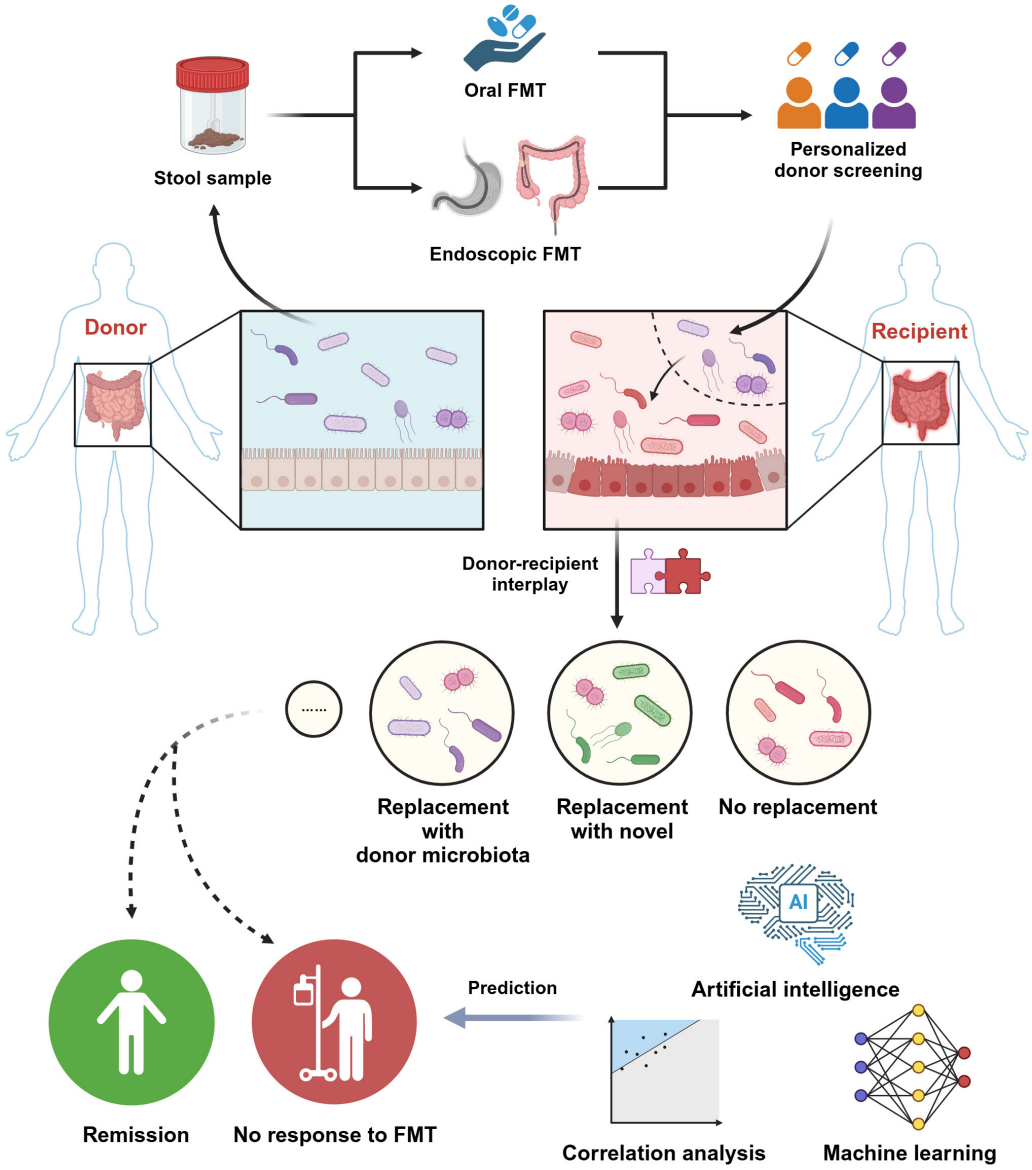
Overview of FMT process, possible ecological scenarios within the gut of recipients post-FMT and the prediction of therapeutic efficacy.

As a therapy based on gut microbiome, the introduction of donor-derived microorganisms would lead to the alteration of recipients' microbiota (Figure 1). The trend of alterations in microbial community is influenced by the baseline microbial features within donor feces and recipient intestine, including the relative abundance of certain bacteria at phylum, family and genus taxonomic levels, and the diversity of gut microbiota, which is generally accessed by alpha-diversity and beta-diversity. Alpha-diversity refers to the richness and evenness of the microbial community, and beta-diversity is defined as the compositional dissimilarity among the microbiome community (Barandouzi et al., 2020). Moreover, the microbial interaction between new colonizers and resident microbiota in the gut of the recipient plays an essential role in the determination of FMT outcome. It is crucial to understand the general patterns of microbial succession following FMT. Generally speaking, the process of the succession of gut microbiota toward the ideal state is supported by the administration of supraphysiologic numbers of strains per species, which increases recipient strain richness, which then gradually converges back to the population average over time after dosing is ceased. In recipients, there is a significant correlation between the strain richness 8 weeks and 5 years post-FMT, showing the durably effect of FMT on the microbial structure of recipients (Chen-Liaw et al., 2024). Simply put, timing is a significant determining factor in FMT.

The ecological scenarios post-FMT in recipient's gut includes no replacement, replace with novel, replace and retain, temporary replacement, gain and retain, temporary gain, and loss of strain. Recipient strains with lower initial relative abundances were more susceptible to replacement than strains that were at higher initial relative abundance (Wilson et al., 2021). Engraftment of strains from relatively abundant species were more likely, and predicted oral, oxygen-tolerant, and gram-positive species had a reduced chance of engraftment in FMT, indicating the importance of microbial adaptation to the gut environment (Podlesny et al., 2022). Another study proved that there was a greater possibility of colonizing in the gut of recipients if a species is among the baseline microbiota of recipients (Li et al., 2016). The dissimilarity in the baseline gut microbiota of donors and recipients is negatively-related with the impact on gut microbiota structure and benefits post-FMT (Benítez-Páez et al., 2022). Loss of baseline strains indicated the niche replacement and out-competition induced by the engraftment of exogenous strains. Interestingly, there is also a study showing that the engraftment of strains from donors follows an “all or nothing” way, which means that the strains are either completely maintained or completely replaced by donor strains post-FMT (Smillie et al., 2018). After FMT, the gaining of new strains which recipients did not possess at baseline occurred more frequently than strain replacement (Wilson et al., 2021). The low baseline SR of species in the gut of recipients would lead to the replacement of recipient strains by therapeutic strains of the same species or engraftment failure, while high baseline strain richness of species provides a microbiota that is more conducive to the engraftment of the same species without replacement (Chen-Liaw et al., 2024). The post-FMT novel strains which could not be detected both in the baseline microbiota of donors and recipients showed strain instability, which may be due to the influence of the environment, the limited detection threshold and undetected secondary strains (Wilson et al., 2021). Inter-individual oscillation could be observed for the appearance of either donor or recipient fecal strain dominance, thus leading to various outcomes in different individuals post-FMT, providing new insights into the dynamics of the microbial community interactions with the recipients post-FMT (Koo and Morrow, 2022). Special microbial interactions can also be detected post-FMT. Recombination could occur between the donor and recipient strains (Koo and Morrow, 2022). Horizontal gene transfer (HGT) is an agent of adaptive evolution enabling the transmission of DNA outside of direct ancestral lineages. FMT does not influence the basal rate of horizontal gene transfer (HGT), which means the transmission of DNA outside of direct ancestral lineages (Behling et al., 2024).

Following the aforementioned patterns, the long-term re-construction of microbiota is performed, and the identities of elevated and decreased strains could be analyzed after FMT, which will be profoundly discussed in the following sections.

## 4 Characteristics of gut microbiota community pre- and post-FMT in various diseases

As have been mentioned above, FMT therapy has been widely used in various diseases associated with microbial dysbiosis. Furthermore, it should be noticed that these diseases exhibit different characteristics of the baseline microbial community, which are generally manifested as the alteration of the abundance of certain strains, or the overall impairment of microbial community after the long-term re-construction of microbiota post-FMT. Mechanistically, the changes in gut bacterial ecology post-FMT can correct the microbiota disorder from the impaired baseline state in certain diseases. For example, FMT can reduce multidrug-resistant organism (MDRO) colonization in a conspecific strain competition manner (Woodworth et al., 2023). Moreover, changes in signaling pathways may be caused by the variations in specific strains.

Considering these specificities, FMT should be more targeted and personalized. Therefore, it can be preliminarily inferred that microbial characteristics post-FMT that more resemble those of healthy donors, along with the restoration of specific microbial members, may have profound influence in disease recovery. Here, we summarize the baseline impairment and re-construction of microbial community observed in patients with FMT-targeting diseases, providing a method for the evaluation of therapeutic efficacy in diseases. Moreover, we introduce the alteration of key molecular expressions and metabolic characteristics in the onset and recovery of microbial disorder-related diseases, revealing the value of FMT at a mechanistic level and laying a foundation for future improvement of therapeutic options.

### 4.1 CDI

CDI can be classified into subtypes including severe CDI and fulminant CDI (FCDI), whose definitions have been stated in ESCMID guidelines and IDSA guidelines (Yakout et al., 2024). In addition, rCDI is defined as a relapse of CDI within 8 weeks of treatment or at least two episodes of severe CDI with hospitalization and significant morbidity (Levy et al., 2024).

16S rRNA gene sequencing showed that the dysbiotic state in rCDI patients is characterized by a large expansion of *Proteobacteria* (Weingarden et al., 2014). A review exploring the link between gut microbiota and CDI development showed that hospitalized elderly individuals with CDI had significantly lower abundances of Lachnospiraceae, Ruminococcaceae, Blautia spp., *Prevotella spp*., Dialister spp., Bifidobacterium spp., Roseburia spp., Anaerostipes spp., *Faecalibacterium spp*. and *Coprococcus spp*., compared with healthy controls, and higher *Enterococcaceae* and *Enterococcus spp.*. While asymptomatic colonization (AC) patients with *C. difficile* showed decreased abundances of *Prevotella, Alistipes, Bacteroides, Bifidobacterium, Dorea, Coprococcus*, and *Roseburia* (Martinez et al., 2022). Another study showed that the relative bacterial abundances of *Negativicutes* (*Firmicutes*), *Gammaproteobacteria* (*Proteobacteria*), and *Fusobacteria* (*Fusobacteria*) were high in CDI patients (Fujimoto et al., 2021).

After performing FMT, increased *Bacteroidetes* and decreased *Proteobacteria* were observed in CDI patients (Seekatz et al., 2014). Another study also proved the importance of *Bacteroidetes* abundance in CDI treatment, indicating that FMT is related with the increased *Bacteroidetes* to *Firmicutes* ratio (Weingarden et al., 2014). *Bacteroidetes* and *Actinobacteria* species showed higher engraftment than *Firmicutes* in recipients' feces in rCDI treatment. Another study showed that the proportions of *Clostridia* (*Firmicutes*), *Erysipelotrichia* (*Firmicutes*) and *Bacteroidia* (*Bacteroidetes*) increased significantly, the richness and diversity of the bacterial species were significantly higher in recipients than those before FMT, and the *bacteriomes* of the recipients tended to approach those of the donors after FMT (Fujimoto et al., 2021). Genomic analyses also showed longitudinal persistent enrichment of *Trichosporonaceae* and *Ruminalococcaceae* bacteria after FMT in CDI patients (Gupta et al., 2016; Ramos et al., 2022). In addition, in a single-center study, an increase in the normal abundance of *Mycobacterium avium* and *Mycobacterium thick-walled* and a decrease in *Mycobacterium aspergillus* were found in the feces of rCDI patients treated with FMT, along with a reduction in the number and diversity of antimicrobial resistance genes in the feces of the patients (Millan et al., 2016).

Functionally, fecal samples from rCDI patients pre-FMT contain high concentrations of primary bile acids and bile salts, instead of secondary bile acids, while post-FMT fecal samples contain mostly secondary bile acids. The identification of bacterial and viral gene functions pre- and post-FMT also revealed improved secondary bile acid biosynthesis, which inhibit the germination of C. difficile spores (Fujimoto et al., 2021; Gupta et al., 2016; Ramos et al., 2022; Weingarden et al., 2014). Enhanced fluorobenzoate degradation can also be detected in rCDI patients post-FMT (Fujimoto et al., 2021). A recent study constructs a mice model and shows that the interaction between *C. difficile*, intestinal commensal microorganisms and the host immune system via inter-related arginine-ornithine metabolism influences the pathogenesis of CDI and the improvement provided by FMT (Yang et al., 2024). Microbial interaction patterns influencing *C. difficile* growth and toxin production is influenced by different nutrient landscapes within the intestine of CDI patients, which helps clarify the therapeutic effect of FMT and improve the effectiveness of anti-CDI strategies (Sulaiman et al., 2024).

### 4.2 IBD

The alpha and beta diversities of microbial populations at the strain level was significantly reduced in UC subjects compared to control. A trail published in Nature followed 132 patients with IBD for 1 year and the increase in facultative anaerobes at the expense of obligate anaerobes and molecular disruptions in microbial transcription (for example, among *Clostridia*) were observed in this study (Lloyd-Price et al., 2019). A recent integrative analysis drew a conclusion that the relative abundance of 117 strains were significantly different between UC and control microbiomes, manifested as decreased *Faecalibacterium prausnitzii* and increased *Ruminococcus gnavus* in UC microbiomes (Zhu J. et al., 2024).

When performing FMT, high bacterial richness and high alpha-diversity in donor fecal is linked with the efficiency of FMT in UC patients (Kump et al., 2018; Rees et al., 2022). The presence of *Bacteroides* (Paramsothy et al., 2019; Rees et al., 2022), *Clostridium clusters IV* and *XIVa* (Paramsothy et al., 2019; Rees et al., 2022), *Akkermansia muciniphila* (Kump et al., 2018), *unclassified Ruminococcaceae* and *Ruminococcus spp*. (Kump et al., 2018; Zhang Z. et al., 2024), *Bifidobacterium* (Nishida et al., 2017) and *Lachnospiraceae* (Moayyedi et al., 2015) in donor stool are correlated with the remission in FMT recipients. The feces of “super-donor” in UC treatment was enriched in *Ruminococcaceae* and *Lachnospiraceae* families (Moayyedi et al., 2015). Whereas *Streptococcus* species (Paramsothy et al., 2019), *Lactobacillales* (Nishida et al., 2017) in donor stool are linked to a lack of response to FMT.

In terms of the FMT recipients with UC, patients in remission post-FMT have enrichment of *Eubacterium hallii, Roseburia inulivorans* (Paramsothy et al., 2019), *Ruminococcaceae* and *Lachnospiraceae* (Pinto et al., 2024), and *Clostridium clusters IV* and *XIVa* compared to patients with no remission post-FMT. Conversely, *Fusobacterium gonidiaformans, Sutterella wadsworthensis, Escherichia* species and *Prevotellaceae* in patients are associated with poor alleviation of UC post-FMT (Paramsothy et al., 2019; Pinto et al., 2024; Wilson et al., 2019). The relapse and poor sustained response of UC post-FMT is correlated with *Proteobacteria* and *Bacteroidetes* (Fuentes et al., 2017). Nevertheless, there are also paradoxes when investigating the significance of microbiota in donor feces, for example, higher *Clostridium clusters IV* level was also observed in the donor feces for nonresponders (Nishida et al., 2017).

Functionally, a cross-cohort integrative analysis enrolling 9 metagenomic and 4 metabolomics cohorts of IBD from different populations proves that essential gene of “Two-component system” pathway, linked to fecal calprotectin, is related with IBD. Moreover, metabolomics analysis shows 36 identified metabolites with significant differences in IBD, and highlights gut microbial biotransformation deficiencies and significant alterations in aminoacyl-tRNA synthetases in IBD patients (Ning et al., 2023). Overabundance of proteases originated from *Bacteroides vulgatus* is associated with UC, which was proved via multi-omics approach (Mills et al., 2022). Another multi-omics study identified impaired metabolism of acylcarnitines, bile acids, SCFAs and levels of antibodies in host serum during IBD activity (Lloyd-Price et al., 2019). The reduction of *Faecalibacterium prausnitzii* and increase of *Ruminococcus gnavus* in UC patients leads to the unique mode of metabolic pathways, characterized by attenuated glycan degradation, fermentation and amino acid metabolism subsystems, while increased citric acid cycle, simple sugars, lipid metabolism, and vitamin and cofactor metabolism subsystems (Zhu J. et al., 2024). For IBD diagnosis, bacterial-associated metabolites including SCFAs, medium-chain fatty acids, tryptophan-derivatives, bile acids and sphingolipids are regarded as metabolism-related biomarkers in clinical practice (Vich Vila et al., 2024). A recent study searches for biomarkers of UC via machine learning and metabolomics, showing the difference of serum levels of tridecanoic acid, pelargonic acid and asparaginyl valine in different subtypes of UC (Ge et al., 2025). In terms of FMT treatment, sustained remission of IBD is associated with overall increased butyrate production capacity (Fuentes et al., 2017), while IBD patients who did not achieve remission post-FMT shows increased levels of heme and lipopolysaccharide biosynthesis (Paramsothy et al., 2019). The baseline levels of xanthine and oleic acid in recipients with IBD are significantly lower than that of the donors and increase after FMT, and putrescine and 5-aminovaleric acid are lower post-FMT compared (Nusbaum et al., 2018).

### 4.3 IBS

IBS could be classified into four subtypes: IBS with predominant constipation (IBS-C), IBS with predominant diarrhea (IBS-D), IBS with mixed bowel habits (IBS-M) or IBS, unsubtyped (Lacy and Patel, 2017). Dysbiosis of intestinal microbiota plays an important role in the pathogenesis of IBS. In a randomized, double-blind, placebo-controlled trial published in Gut that included 52 adult patients with moderate to severe IBS, 11 OTUs established in FMT recipients, 6 of which were classified in the *Clostridiales* order and 4 of which were classified in the *Bacteroidales* order. The IBS-SSS was negatively-correlated with *Blautia* genus of the *Clostridiales* order, which is associated with a healthy gut-microbiome (Halkjær et al., 2018). *Dorea, Lactobacillus* and *Ruminococcaceae spp*. in recipients' feces are associated with higher success rate of FMT when treating IBS (El-Salhy et al., 2020).

The characteristics of baseline microbiota impairments in IBS subtypes differ. In a meta-analysis of a randomized controlled study showed that IBS-C patients had higher fecal *Bacteroides* level, while no significant increase in *Bifidobacterium, Lactobacillus, Enterobacteriaceae*, or *Enterococcus* were found (Shukla et al., 2015). In another randomized controlled study enrolling 27 individuals with IBS-D, 7 bacterial genera (*Gemella, Roseburia, Acidovorax, Lactobacillus, Weissella, Klebsiella* and *Parvimonas*) were associated with differences in the pre- and post-treatment IBS-SSS score, and *Gemella, Acidovorax* and *Klebsiella* might be involved in the development of the clinical symptoms of IBS-D (Zhang Y. et al., 2024). The level of *Faecalibacterium prausnitzii* has a potential relation with IBS-M (Soldi et al., 2019).

For the mechanism, in IBS patients, the links between gut microbiota and fecal metabolites are observed, revealing that *Odoribacter splanchnicus, Escherichia coli* and *Ruminococcus gnavus* are strongly associated with the low abundance of dihydropteroic acid. Furthermore, tryptophan/serotonin metabolism disorder is related with IBS depression comorbidity (Han et al., 2022). Through analyzing the longitudinal multi-omics data of IBS patients, the potential effect of purine metabolism associated with microorganisms in IBS is identified (Mars et al., 2020). The global alterations in microbiome composition in IBS patients also result in increased tyramine, upregulation of fructose and glucan metabolism, succinate pathway of carbohydrate fermentation, and decreased gentisate and hydrocinnamate. Moreover, IBS-D and IBS-C shows different characteristics in the metatranscriptome and metabolome, implying the importance of focusing on the subtypes of diseases (Jacobs et al., 2023). DESI-MSI shows that 6 medium-chain and long-chain fatty acids are determined to be most overrepresented in the IBS-D group, becoming potential indicators to distinguish IBS patients and healthy population (Zhang Y. et al., 2023). FMT increases the fecal SCFA levels in IBS patients, which are related with improved clinical symptoms of patients (El-Salhy et al., 2021).

### 4.4 Other potential FMT-targeting diseases

In metabolic diseases, successful FMT has been shown to significantly increase the abundance of SCFA-producing species such as *Roseburia intestinalis* and *Akkermansia muciniphila*, as well as various *Clostridium spp* (Kootte et al., 2017). In the treatment of severe obesity and metabolic syndrome, lower relative abundance of *Prevotella*, greater bacterial richness and more consistent engraftment of donor-specific bacteria ASVs (amplicon sequence variants) are associated with better treatment response (Zhang Z. et al., 2024). In obesity treatment, multi-donor FMT showed the efficiency of sustainably altering the microbiome of recipients, with 2 of the 4 donors dominating the microbial engraftment to the recipient. Exploring the gut microbiome characteristics of the two primary microbial strain providers, results showed high ratio of *Prevotella* to *Bacteroidetes* (P/B) dominated the engraftment and almost all FMT recipients with a low P/B ratio at baseline transitioned to a high P/B ratio (Wilson et al., 2021).

In immune-related diseases, a non-randomized clinical trial enrolling 10 individuals with immune-mediated dry eye, after administration of FMT in two enemas one week apart, subjects were found to have decreased abundance of *Enterococcus faecalis spp., Prevotella spp*., and *Ruminalococcus spp*., and increased abundance of the genera *Alistipes, Streptococcus*, and *Blautia*, as compared to the donors (Watane et al., 2022). In GvHD treatment, an increase in the richness and diversity of the intestinal bacterial group was found in subjects with GvHD who received two consecutive FMTs, and three major species were detected in the subjects' feces, including *Alistipes putredinis, Clostridium nexile*, and *Ruminococcus gnavu* (Zhang et al., 2021).

In neurologically related disorders, for example, when treating autism spectrum disorders (ASD), decreased *Collinsella* level is found in responded FMT recipients, and the relationship between other common bacterial strains and the treatment effect has also been elucidated (Chen et al., 2024). Meanwhile, in ASD patients, the pre-treatment abundance of *Eubacterium coprostanoligenes* was lower in responders, which was also negatively correlated with the improvement of gastrointestinal symptoms and the concentration of serum γ-amino acid (GABA), indicating its potential modulating role in the treatment of ASD by FMT (Li et al., 2021).

## 5 Definition of successful FMT: microbial engraftment vs. clinical outcomes

The characteristics of microbial changes in patients who successfully recovered from various disease, as listed above, suggest that we can to some extent assess the efficacy of FMT by analyzing the microbiota of recipient post-FMT. The changes which meet expectations in the recipient's gut microbiota following FMT can provide optimistic signals in a success therapy to some extent.

Generally, researchers define the success of FMT as the shift in the gut microbiome profile of recipient toward that of the donor and further augmentation of the local commensal community (Wilson et al., 2019). However, fundamentally, as a clinical therapeutic approach, it is the restoration of health after treatment that become the most important criterion for demonstrating the value of FMT. As expected, some studies suggest that considerable engraftment of donor strains is equivalent to high therapeutic efficacy in FMT. Donor microbial profile similarity in recipient post-FMT can be regarded as a predictor at response (Rees et al., 2022), for example, the microbiota of responders post-FMT was similar to that of their healthy donors in UC treatment (Rossen et al., 2015). Moreover, if we could identify the patient's impairment of microbiota and metabolic characteristics which are specific to their disorder, the donor-recipient matching approach may efficiently help recipients in reconstructing disrupted physiological conditions in a targeted manner (Wilson et al., 2019). As a result, ulteriorly, the screening of donors with certain structure of microbial community may help improve the efficacy of FMT.

Recently, the screening of “super-donor” before FMT is becoming more and more popular, which points to donors whose stool samples results in significantly more successful FMT outcomes than that of other donors (Wilson et al., 2019). In a randomized controlled trial of patients with obesity, multi-donor FMT was able to sustainably alter the patient microbiome, with two of four donors dominating the microbial engraftment of the recipient, which were characterized by high *Prevotella* to *Bacteroidetes* ratio, showing the tendency toward being a super donor (Wilson et al., 2021). In a mice gut colonization model, researchers identified a super-donor consortium, which could effectively induce the engraftment of microbiota into recipients. In FMT induced by these super-donors, we could observe a rapid engraftment by early colonizers within 72 h, followed by a slower engraftment by late colonizers over 15-30 days. Spatial transcriptomics has revealed that the microorganisms introduced into recipients are distributed in distinct niches over time, which partially summarized the mechanism of super-donor colonization (Urtecho et al., 2024). In a randomized controlled study, donor-recipient-matched FMT significantly improved the clinical symptoms, quality of life and anxiety scores of the patients with IBS-D than random-donor FMT (Zhang Y. et al., 2024). In short, the screening for super-donors adheres to the fundamental principle of microbiota modulation, and is directed toward achieving better clinical outcomes.

However, some studies did not support the necessary connection between microbial engraftment and clinical efficacy. A randomized controlled trial performed rigorous donor selection based on microbial cell count, enterotype and the abundance of specific genera. Unexpectedly, the trail has been halted for futility (Caenepeel et al., 2024). Moreover, although the persistent engraftment of strains from selected donors has been detected in the recipient, it might also end in a failed treatment, the engraftment of fecal dominant donor microbes of the donor is not necessarily correlated with clinical improvement (Browne et al., 2021; Koo and Morrow, 2021).

Therefore, a successful FMT should not be merely defined as the influence of the donor and the alterations in the gut microbiota. The exact mechanisms of “why FMT works” still remains to be explored in future researches.

## 6 Objective assessment tools for FMT efficacy: prognostic models

As mentioned above, donor screening based on microbiota characteristics may not necessarily lead to better prognosis, implying the presence of potential determining factors. In addition, due to the specificity of intestinal microecology, the complex interplay between microbial members leads to the difficulty to be exactly identified and included in the strategy of donor screening. Therefore, the construction of prognostic models could provide us with comprehensive insights into the factors influencing FMT efficacy. Specially, through the mining and analysis of a vast amount of data, machine learning algorithms are capable of precisely capturing the intricate correlations among FMT and disease treatment outcomes, thus providing novel perspectives and methodologies for disease diagnosis, treatment, and prognosis evaluation.

Wei et al. successfully fitted a random forest model to predict the treatment outcome 8 weeks post-FMT in rCDI patients, in which the factors that have the greatest impact on the output of the model are the abundance of *Enterobacteriaceae* and *Lachnospiraceae* at week 1 in recipients (Wei et al., 2022). Another study's application of random forest modeling in patients with rCDI found that bacterial abundance, classification and time after FMT were important predictive factors (Smillie et al., 2018). Besides random forest models, a regression tree-based model could effectively predict the outcome (response and recurrence) of cap-FMT in treating rCDI, which includes taxa significantly related to clinical response (Staley et al., 2018). While Xiao et al. present an ecological framework using rCDI as a prototype disease to predict the taxonomic diversity of the diseased state, the impact of host-dependent microbial dynamics, and each level of host-dependency of the microbial dynamics on FMT efficacy, which provides innovative ideas for follow-up research (Xiao et al., 2020). Using metagenomic sequencing and machine learning, Kazemian N et al. proved that in rCDI treatment, the presence of baseline *Clostridioides spp., Desulfovibrio spp., Odoribacter spp*. and *Oscillibacter spp*., etc, in donors and the absence of baseline *Yarrowia spp*. and *Wigglesworthia spp*. in recipients prior to FMT could predict FMT success (Kazemian et al., 2020).

In UC patients, Sood A et al. develpoed a nomogram predicting the response to FMT, which was defined as the achievement of corticosteroid free clinical remission at week 30. The factors associated with clinical remission includes younger age, disease extent E2 and endoscopic mayo score 2 (Sood et al., 2020). A LASSO logistic regression model was constructed using *Enterococcus, Rothia*, and *Colidextribacter* as predictors of UC response, and the AUC of the constructed model amounted to 0.84 (Kang et al., 2022). On the prediction of FMT treatment outcomes in patients with UC, Wu X. et al. utilized the random forest classifier to construct a model based on 20 serum metabolites screened by the Boruta method, in order to predict the clinical remission of UC patients after FMT treatment. The analysis revealed that the model had good predictive performance, with an AUC of 0.963, and good performance in terms of accuracy, sensitivity, and specificity (Wu X. et al., 2023). Zhang S. et al. used multiple machine learning models to construct an integrated model for predicting the clinical response of UC patients after one month of WMT treatment. After internal and external validation, it was found that the different integrated models had their own advantages and disadvantages, and that the vector machine was more stable and reliable (Zhang S. et al., 2024). Zou et al. applied the random forest algorithm and used its classification model to predict metagenomic OUT linkage groups (mOTU) presence and regression model to predict mOTU abundance, it can predict the gut microbiota composition of post-FMT recipients (Zou et al., 2020). Another machine learning model can predict the presence or absence of strains in recipients post-FMT in UC recipients (Ianiro et al., 2022).

To track strains in FMT, Aggarwala et al. assembled a collection of over different bacterial strains from the fecal samples of 22 FMT donors and recipients, and further developed a statistical approach named Strainer, which, in combination with culture and sequencing data, was rigorously benchmarked to accurately detect and quantify the colonization of donor strains in recipients and the retention of original strains in recipients (Aggarwala et al., 2021). To predict the outcome of FMT transplantation, the iMic algorithm was developed, based on the microbiological characteristics of human fecal donor samples, combined with multiple machine learning models, and evaluated with multiple metrics after data preprocessing. iMic was found to perform well in predicting the microbiome characteristics of the recipients and the clinical outcomes, and the demographic information of the donors could improve the prediction results (Shtossel et al., 2023). To investigate how donor-derived bacteria affect FMT efficacy in both CDI and IBD patients, He R et al. recruited 2 longitudinal IBD cohorts of 103 FMT recipients and further analyzed 1,280 microbiota datasets from 14 public CDI and IBD studies. This research propose a new parameter defined as the ratio of colonizers to residents after FMT (C2R) to evaluate the engraftment of donor microbiota in recipients. An enterotype-based donor selection (EDS) statistical model based on enterotype (RCPT/E dominated by *Enterobacteriaceae* and RCPT/B dominated by *Bacteroides*) has been constructed to predict the level of donor-recipient matching (He et al., 2022). More comprehensively, Schmidt TSB et al. analyzed metagenomes from 316 FMTs for the treatment of 10 different disease indications, thus constructed a LASSO-regularized regression model that could predict the recipient strain turnover, recipient resilience, donor colonization and donor takeover with considerable accuracy. In this study, more clearly, the variables could be divided into ex ante variables, which are knowable pre-FMT, and *post hoc* variables, which are measurable post-FMT (Schmidt et al., 2022). On the whole, the above-mentioned researches extended the prediction model to various diseases, in other words, broke down the barriers between diseases and proposed a set of universal conclusions about the microbial community post-FMT, challenging the hypothesis of “super donor” and underscoring the importance of a multifactorial prediction of FMT efficacy, including recipient factors.

In short, current studies have achieved remarkable results in predicting the treatment efficacy of FMT for diseases related to the gut microbiota. The application of various algorithms, ranging from logistic regression-based nomogram models, machine learning models and newly developed statistical approaches or algorithms, has continuously deepened our understanding of the relationship between the microbiota and FMT (Table 1). By customizing the most optimal donor for each recipient, it provides strong support for precision medicine. In the future, mechanisms of the regulatory effects provided by the factors involved in the prediction models will be revealed, enhancing the explainablity and transparency of predictive models.

**Table 1:** Prediction models for donor microbiota engraftment and clinical outcomes in FMT treatment.

**References**	**Type of the prediction model**	**Disease under investigation**	**Population enrolled**	**Dominant predictors of the model**	**Prediction target**	**Area under the curve (AUC)**
Wei et al. (2022)	Random forest model	rCDI	64	The taxa at week 1 post-FMT at genus level • Enterobacteriaceae (Escherichia, negatively correlated with response) • Lachnospiraceae (Blautia, positively correlated with response) • Ruminococcaceae	Treatment outcome at week 8	0.79
Smillie et al. (2018)	Random forest model	rCDI, metabolic syndrome	19-rCDI 5-metabolic syndrome	•The abundance of bacteria • The phylogeny of bacteria	Composition of the gut microbiota in patients after FMT	0.84-rCDI 0.82-metabolic syndrome
Staley et al. (2018)	Chi-squared automatic interaction detection (CHAID)-based regression tree model	rCDI		The abundances of members of the families at 7 days after cap-FMT • Lachnospiraceae • Ruminococcaceae • Bacteroidaceae • Porphyromonadaceae • Enterobacteriaceae	The eventual recurrence of CDI following cap-FMT	
Xiao et al. (2020)	Lotka-Volterra model	rCDI		•The impact of host-dependent microbial dynamics • Each level of host-dependency of the microbial dynamics • Taxonomic diversity of the diseased state	The FMT efficacy	
Kazemian et al. (2020)	Random forest model	rCDI	17	The gut microbiome of donor and recipients pre-FMT (1 week prior to FMT); Desulfovibrio, Filifactor, Bacillus, Yarrowia, Odoribacter, Wigglesworthia, Oscillibacter, Intestinimonas and Clostridioides	Treatment outcome	0.98
Sood et al. (2020)	Logistic regression-based nomogram	Active UC	93 patients who completed the multi-session FMT protocol	•Age • Disease severity • Disease duration • Disease extent • Endoscopic mayo score	Response to FMT (achievement of corticosteroid free clinical remission at week 30)	
Kang et al. (2022)	LASSO-regularized regression model	UC	10	•Enterococcus • Rothia • Colidextribacter	Successful response (defined as partial Mayo score and CRP reduction)	0.84
Wu X. et al. (2023)	Random forest model	UC	44	20 metabolic markers • Glycerophosphocholines • Glycerophospholipids • Glycerophosphoethanolamine	Clinical remission after 3 months of FMT in UC patients	0.96
Zhang S. et al. (2024)	Voting machine Logistic regression Random forests Adaptive Boosting Light gradient boosting machine Support vector machine	UC	366 (210-training and internal validation; 156-external validation)	•Age • Defecation frequency • Mayo score • Platelet distribution width • Platelet Large Cell Ratio • γ-glutamyl transpeptidase	Clinical response	0.78; 0.614- external validation
Zou et al. (2020)	Random forest model	IBD	15	•The presence of each mOTU • The abundance of each mOTU	Gut microbiota mOTU profiles	0.74
Ianiro et al. (2022)	Random forest model			•Taxonomy • Microbial abundances • α-diversity • Microbial prevalence	The presence or absence of Species post-FMT	0.77
Aggarwala et al. (2021)	Strainer (Statistical methods)	rCDI	13	•Types of strains • Number of strains	Proportional Engraftment of Donor Strains(PEDS) Proportional Persistence of Recipient Strains(PPRS)	0.86
Shtossel et al. (2023)	iMic (image microbiome) Random forest model	IBD, CDI, IBS		•Microbiome characterization of the donor • Donor demographics: Age, sex, weight	Microbiome Characterization of Post-FMT Recipients Improvement in clinical symptoms	0.71-WGS cohorts 0.69-the 16S cohort 0.71-recipient-based learning 0.71- combined donor and recipient model
He et al. (2022)	Random forest model	IBD, CDI	286	•Enterotypes of recipients • Enterotypes of donors before FMT • Their corresponding microbial profiles	Outcome of FMT for each Recipient-donor pair	0.80
Schmidt et al. (2022)	LASSO-regularized regression model	rCDI, ESBL, MetS, IBD, etc	316	•Recipient resilience • Donor colonization • Donor takeover • Recipient strain turnover	Strain dynamics after FMT	0.62-recipient resilience 0.58-donor colonization 0.65-donor takeover 0.94-recipient strain turnover

## 7 New players of microorganisms other than bacteria in FMT

As the development of research on the transplantation of the virome and fungal to recipients, the significant role of these new players other than bacteria in human health and the FMT process is gradually being unveiled.

The human gut virome is dominated by bacteriophages, which are a major component of the human gut microbiota. Emerging evidence suggests that gut bacteriophages play important roles in the intricate dynamics with bacteria and their transfer may be associated with the efficacy of FMT. Bacteriophage transfer might also be of substantial mechanistic importance in FMT because of their ability to maintain microbiome ecology equilibrium with bacteria (Liu et al., 2022). In the study of FMT for the treatment of patients with rCDI, successful FMT donors were found to have higher phage alpha diversity and lower relative abundance, suggesting that FMT with increased phage alpha diversity is more likely to successfully treat rCDI (Park et al., 2019). In a longitudinal study of healthy subjects and obese subjects treated with FMT, recipients with improved clinical outcomes had phage communities that shifted significantly toward healthy donors, with high abundance of phages HV39 and 84 associated with increased rates of glucose disappearance and better clinical outcomes (Manrique et al., 2021). Macrogenomic sequencing showed that FMT altered the characteristics of the enterovirome in post-FMT recipients compared to pre-FMT. For example, the proportion of microviridae in the recipients increased, and the behavior of most temperate phages paralleled that of host bacteria altered by FMT (Fujimoto et al., 2021). Fecal virome transplantation (FVT) has been applied in certain clinical contexts, however, it carries the risk of eukaryotic viral infections. Therefore, modified FVT characterized by removed or inactivated eukaryotic viruses in the viral component, as a efficiency method of safe bacteriophage-based therapies (Mao et al., 2024). Taken together, these findings highlight the association between gut phage and the clinical success of FMT therapies for various diseases. Recently, a growing number of studies have identified *Caudovirales phages* that may interact with host microorganisms and influence clinical outcomes after FMT. Accumulating data has demonstrated that *Caudovirales phages* may play a role in the efficacy of FMT in different diseases. CD and UC were found to be associated with significant amplification of *Caudovirales phages* (Fujimoto et al., 2021). Additionally, *Caudovirales phages* were found to be reduced in UC patients who responded to FMT compared to those who did not respond to the treatment (Gogokhia et al., 2019). Similarly, in a clinical trial, subjects with CDI demonstrated a significantly higher abundance of *Caudovirales phages* and a lower *Caudovirales* diversity, richness and evenness compared with healthy household controls. FMT treatment resulted in a significant decrease in the abundance of *Caudovirales* in CDI (Zuo et al., 2018a). Contrary to the findings of the previous two diseases, in a single case study exploring the use of FMT in a patient with severe gut GvHD, an increase in fecal virome diversity was observed after FMT, accompanied by increased *Caudovirales phages* and a reduction in the relative abundance of eukaryotic Torque teno viruses (Zhang et al., 2021).

Changes in fungal composition have also been associated with various diseases. In IBD patients, a decrease in *Saccharomyces cerevisiae* and *Filobasidium uniguttulatum* species and an increase in *Candida* (e.g., *Candida albicans*) were observed relative to healthy controls (Liguori et al., 2016). The presence of pre-FMT fungi (e.g., *Candida*) was also been found to be associated with increased bacterial diversity after FMT in UC patients (Leonardi et al., 2020). In rCDI patients, successful FMT was dependent on high abundance of *Saccharomyces* and *Aspergillus*, while *Candida* was negatively associated with successful FMT (Zuo et al., 2018b), in contrast to the results observed in UC patients (Leonardi et al., 2020). In the metagenomic analysis of fecal samples from donors and patients with UC receiving capsulized FMT, shifts in gut fungal diversity and composition were associated with capsulized FMT and validated in patients with active UC. Decreased levels of pathobionts, such as *Candida* and *Debaryomyces hansenii*, were associated with remission in patients receiving capsulized FMT (Chen et al., 2022).

## 8 Emphasizing FMT safety: focusing on infection sources and susceptible populations

### 8.1 Transmission of pathogens associated with donor feces

Despite its increasing popularity as a therapeutic intervention, FMT still faces numerous regulatory and safety challenges. Just as the engraftment of donor microbiota, potential pathogens may also be transmitted to the recipient's gut. MDROs including extended-spectrum beta-lactamase (ESBL)-producing *Escherichia coli* (*E. coli*) can be detected in some of donor feces and a quarter of active donors were colonized with MDROs during participation in the donor programme (Vendrik et al., 2021). It was reported that two patients developed bacteremia due to ESBL-producing *E. coli* after receiving stool from the same donor for FMT and one of the patients died, emphasizing the importance of minimizing the transmission of potentially pathogenic microorganisms, thereby reducing the risk of adverse infectious events (DeFilipp et al., 2019). Stool donor colonized by Shiga toxin-producing *E. coli* (STEC) can also lead to adverse events after FMT (Zellmer et al., 2021). It is worth noting that the fecal carriage rate of ESBL-producing *Enterobacterales*, including *E. coli* and *Klebsiella pneumoniae*, and *diarrheagenic E. coli*, including EPEC, *enteroaggregative E. coli, enterotoxigenic E. coli* and STEC, were high (Chuang et al., 2023). Opportunistic infections induced by potential infected donors should be given increasing amount of attention, for the infection of these pathogens may not be symptomatic in IC donors but may lead to transmissible to immunosuppressed FMT recipients (Mehta et al., 2022).

Whole genome sequencing indicated strain transmission of procarcinogenic bacteria between donor and patient, and patients also exhibited clearance of procarcinogenic bacterial strains subsequent to negative donor in FMT (Drewes et al., 2019). Interestingly, a substantial proportion of recipients with potentially procarcinogenic polyketide synthase-negative (pks-) status who underwent FMT from pks+ donors remained pks-, while some pks- recipients treated with stool samples from pks- donors developed pks+ *E. coli* post-FMT (Khoruts, 2021). To sum up, although there is no exact sign of the donor-to-patient transmission of pks+ E. coli, the pks abundance and persistence of pks+ *E coli* of rCDI patients are associated with the pks+ donor (Nooij et al., 2021).

Specially, during the pandemic of infectious diseases, extra detection should be performed to avoid FMT-related transmission of pathogens. During the COVID-19 pandemic, the program of donor recruitment further included evaluation of clinical history and specific testing for the detection of SARS-CoV-2 including nasopharyngeal swab, reverse transcription polymerase chain reaction (RT-PCR) assay, serology and molecular stool testing, etc, showed significant effect in epidemic prevention (Ianiro et al., 2020).

A recent study provided an end-to-end donor screening program, which can effectively minimize the risk to patients receiving FMT. The following factors of donor screening should be valued: health history, physical exam, visual inspection of donations and laboratory testing. Failure modes and effects analysis (FMEA)-based donor screening can be applied to avoid the risk of disease transmission from donors to recipients (Goldsmith et al., 2024).

### 8.2 Balancing the risks and benefits of FMT in immunosuppressed patients

In general, immunocompromised (IC) patients, including patients on immunosuppressant medications, with human immunodeficiency virus (HIV), inherited or primary immunodeficiency syndromes, cancer undergoing chemotherapy or organ transplant, were often excluded from FMT trials (Shogbesan et al., 2018). FMT is contraindicated in patients with significant primary and secondary immunodeficiencies, which is owe to the potential risk of bacterial translocation and the development of bacteremia, particularly with MDROs (Conover et al., 2023). However, FMT probably could be generally considered safe in IC patients in some conditions according to several studies. Research showed similar rates of serious adverse events when using FMT to IC patients compared to those with intact immune function (Shogbesan et al., 2018). When treating pediatric IC patients with rCDI by FMT, the success rate was similar to the treatment targeting IC adults and immunocompetent children (Conover et al., 2023; Rodig et al., 2023). A pilot placebo-controlled study showed that FMT is safe in HIV patients and led to no severe adverse events and attenuated HIV-associated dysbiosis (Serrano-Villar et al., 2021).

Patients in the following cases, who presented with varying degrees of immunosuppression, achieved alleviation of their primary conditions following FMT. A 59-year-old patient with common variable immunodeficiency accepted FMT to alleviate diarrhea after all therapeutic options had been exhausted, which turned out to be successful and led to no adverse effects (Napiórkowska-Baran et al., 2024). Three patients with refractory acute graft-vs.-host disease (GI-aGvHD) following allogeneic hematopoietic stem cell transplantation (allo-HSCT) also achieved sustained improvement after FMT (Spindelboeck et al., 2017). A single heart-kidney transplant recipient with rCDI, vancomycin-resistant *Enterococcus* (VRE) fecal dominance and recurrent VRE infections obtained relief from the above-mentioned infections via FMT (Stripling et al., 2015).

However, while using FMT in IC patients is accepted to a certain degree, we still need to pay more attention to this population to ensure their the safety and curative effect of FMT. From the aspect of success rate, repeated FMT or additional antibiotics may be needed to achieve improved outcomes in solid organ transplant (SOT) patients (Cheng et al., 2019). Most importantly, from the aspect of safety, several reported severe adverse effects of FMT in IC patients emphasizes the potential risks associated with FMT treatment to this typical population. Undetected opportunistic pathogens in stool samples might bring the risk of pathogenic transmission in immunosuppressed patients, including cytomegalovirus (CMV), Epstein Barr virus (EBV) and polyomaviruses, etc. (Mehta et al., 2022). Some researchers insist that although FMT therapy is not associated with the increased risk of severe adverse events according to several studies, the degree of immunosuppression should be accessed prior to the application of FMT, especially for patients with solid tumors receiving cytotoxic therapy, for the type and duration of chemotherapy varies, which may influence the safety and success rate of FMT. Specially, in addition to the function of individual's immune system, mucosal immunity is also an essential part in the resistance to pathogens. It should be noted that all reported FMT-related serious adverse events were observed in patients with mucosal barrier injury (Marcella et al., 2021). More case-by-case assessment of the benefit-to-risk ratio should be performed to guide the protocols of FMT for IC patients and help grasp the potential chances of therapy (Benech et al., 2024).

## 9 The potential influence of gender in the outcome of FMT

Sex differences in incidence rates of multiple diseases has been found, and gender factors are closely associated with the pathogenesis of some diseases. For example, a newly published review has discussed the effect of gender in IBD, which could help advance personalized medicine and improve the quality of life for people with IBD in a gendered point of view (Andersen et al., 2024). As a result, some studies insist that gender should be considered while donor screening to ensure the success of FMT (Benítez-Páez et al., 2022).

It is worth noting that researches have proposed the contribution of gender factors in the outcomes of FMT. Overall, gut microbiota differed in males and females, which could be partially explained by the impact of sex hormones (Yurkovetskiy et al., 2013). Individuals of different sexes possess unique physiological characteristics and less susceptibilities to certain diseases and these factors might be transferred to recipients post-FMT, whose mechanisms may involve that the gut bacterial community composition differs between male and female, and such difference can further modulate the metabolic processes and molecular expressions (Haro et al., 2016). As a result, in several diseases, stool sample from a certain gender might be considered as potential donors with higher quality. Improved outcome of ischemic stroke could be induced by female donor of FMT, for the gut microbiota of female is characterized by lower level of systemic proinflammatory cytokines (Wang J. et al., 2022). The susceptibilities to radiation toxicity (Cui et al., 2017) could also be transferred via FMT in a gender specific manner. During the process of FMT, change in the gender of either the donor or the recipient may lead to different clinical outcomes. The baseline characteristics of microbiota differs in donors of different sex, and the gender of recipients influences the strain colonization, the gut of mice of different genders prefers to accommodate microbiota differently (Wang et al., 2016). Post-inflammatory females with colitis could transfer visceral hyperalgesia to both males and females, but males could only transfer visceral hyperalgesia to individuals of the same sex (Arzamendi et al., 2024). Feces of young female mice could improve insulin sensitivity in females, while feces of aged male mice could increase insulin resistance in female mice, eliminating and enhancing sex differences in insulin sensitivity and metabolome, respectively (Sheng et al., 2021). FMT during lactation resulted in long-term effects on the metabolism of male Wistar rats while no effect was observed in female rats (Pavanello et al., 2022).

In some cases, despite considering the potential benefits that certain gender may provide in FMT, attention must be paid to the risks associated with sex-discordant FMT. A recent research published in Gastroenterology proved that sex-concordant FMT contributes to fewer adverse events post-FMT, and sex-discordant FMT administrated while treating CDI has the potential to cause IBS (Sehgal et al., 2024). Another study showed that male recipients who underwent cross-sex FMT exhibited notably reduced testosterone levels in comparison to those who received same-sex FMT (Feješ et al., 2024), suggesting the disruption of gender-specific baseline physiological characteristics caused by sex-discordant FMT.

In conclusion, as an often overlooked demographic indicator, existing research has proposed the multidimensional impact of age on FMT outcomes, while we have not yet formed a detailed proposal to generalize to clinical applications. More research is imperative in the future.

## 10 The potential influence of age in the outcome of FMT

Gut microbial composition is considered to be associated with the age. Interestingly, a gut microbial age (MA) metric has been proposed to evaluate gut aging (Wang et al., 2024). As a result, the age of either the donor and the recipient may affect benefits provided by FMT due to age-related baseline microbial characteristics.

Generally, we advocate prioritizing young donors in donor screening. According to statements provided in European consensus conference on FMT in clinical practice, individuals aged < 60 years should be preferred while selecting donors, while this age restriction is not must be strictly adhered to, the criteria may be appropriately relaxed under certain circumstances, for instance, the use of intimate healthy partners (Cammarota et al., 2017). In animal experiments, aged donor could lead to age-associated alteration of physiological activities and signaling pathways, resulting in the occurrence of major comorbidities associated with aging. Conversely, the transfer of young donor microbiota to old recipients could reverse aging-related alteration (Cheng et al., 2024; D'Amato et al., 2020; Parker et al., 2022). Aged mice is susceptibility to arrhythmia for the increased reactive oxygen species (ROS) level, which could be transmitted to young mice via FMT (Fu et al., 2024). Nevertheless, strikingly, under some circumstances, the older, the better. An aging-enriched enterotype was observed to conduce to improved immunotherapy outcomes in older patients with cancer, which was characterized by the up-regulation of exhausted and cytotoxic T cell markers in the tumor microenvironment and whose therapeutic effect in FMT has been proved in mice (Zhu X. et al., 2024). However, we still need to consider how this finding can be translated into clinical practice, as other physiological impairment caused by gut aging seem to be difficult to avoid.

The age of the recipient also has the potential to affect the efficiency of FMT. A prospective cohort study proved that patients with rCDI who aged over 65 years might be independently associated with a lower treatment effect from a single FMT (Baunwall et al., 2023). However, in another prospective study, genomic analysis showed that there was no significant difference in gut microbial diversity between donors aged ≥60 years and < 60 years. The clinical efficacy of FMT in rCDI over 12 months was also not affected with advancing age (Anand et al., 2017). According to the second edition of joint British Society of Gastroenterology (BSG) and Healthcare Infection Society (HIS) guidelines (2024), we should not refuse or delay FMT therapy due to any recipient risk factors, for example, recipient age over 75 years old (Mullish et al., 2024).

The age disparity between the donor and the recipient is of significant importance. When treating metabolic syndrome by FMT, the age difference of donor-recipient pairs is positively-related with the greater reversion of metabolic dysfunction (Benítez-Páez et al., 2022). Specially, significant age disparities between the donor and the recipient could occur in the FMT treatment of pediatric intestinal and extraintestinal diseases, it seems that pediatric patients have been also receiving transplant material from adult donors. The differences in gut microbiota between children and adults need to be taken seriously.

Specialized metabolic functions in pediatric recipients are observed while using adult fecal donors (Wu Q. et al., 2023). The match of adult donors and child recipients has the risk of atypical maturation of gut microbiota in pediatric patients (MacLellan et al., 2021). However, it needs to be noted that although there are challenges in the use of FMT in pediatric patients, it is necessary to overcome these difficulties and develop a more widely recognized standard, especially due to that the approvals of promising biotherapeutics are expected to be significantly delayed for children (Hourigan et al., 2021).

## 11 Metabolic status and lifestyle of donors and recipients

The metabolic status of the donor should be evaluated before FMT procedure, which could be regulated via lifestyle intervention. Diet is the main contributor to gut microbiota diversity, the proportional abundance of several taxa varies with the variation of diet patterns and body fat counterpart levels (Newman et al., 2021). Accompanied with the alteration of microbiota, different dietary patterns shape various metabolomic characteristics, manifested as the variation of 127 common metabolites including lipids, tri/di-glycerides and lyso/phosphatidylcholine, etc. (Tanaka et al., 2022). Specially, the Mediterranean diet intervention could lead to increased fiber-degrading *Faecalibacterium prausnitzii* and gene expression for microbial carbohydrate degradation associated with butyrate metabolism, fecal bile acid degradation and insulin sensitivity that co-varied with specific microorganisms (Meslier et al., 2020).

Animal experiments proved that increased total body and fat mass, as well as obesity-associated metabolic phenotypes were transmissible with FMT (Ridaura et al., 2013). Both leptin receptor knock-out obese and diabetic mice donor and diet-induced obese mice donor could result in elevated gut permeability, inflammation level and glucose metabolic dysfunctions in mice recipients, which could be partially explained by the impaired ethanolamine metabolism (Mishra et al., 2023). Donor mice fed by high-fat, high-sucrose diet (HFHS) could induce disrupted glucose metabolism to recipient mice, while secondary adiposity was not observed in recipients (Zoll et al., 2020).

Conversely, donors who have a healthier habitus and adherence to healthy lifestyle are more likely to confer long-term health benefits to recipients. Within 6 weeks after FMT induced by lean donors, FMT resulted in significantly altered duodenal bacterial species including *Bifidobacterium pseudolongum*. Fecal bacterial species that were different between autologous and allogenic FMT from lean donors included the lactate-producing *Lactobacillus salivarius* and butyrate-producing *Butyrivibrio, Clostridium symbiosum*, and *Eubacterium* species, which were related to human metabolism (Kootte et al., 2017). Lean vegan donor could induce improvement of the outcome of recipients with hepatic steatosis via shaping microbial community and altering the expression of hepatic genes involved in inflammation and lipid metabolism (Witjes et al., 2020).

A randomized controlled trial published in Gastroenterology randomly divided abdominally obese or dyslipidemic individuals into three groups, in which participants followed healthy dietary guidelines, Mediterranean diet guidelines and green-Mediterranean diet guidelines (extra consumption of green tea and a Wolffia globosa green shake). After 6 months of weight-loss phase, participants underwent autologous FMT and those who was in green-Mediterranean diet group showed less weight regain, gut microbiota recovery and metabolic impairment. Mechanistically, the green-Mediterranean diet induced altered microbiome composition during the weight-loss phase, and prompted preservation of weight-loss-associated specific bacteria and microbial metabolic pathways including microbial sugar transport after autologous FMT (Rinott et al., 2021). Specially, although stool samples gained from metabolic syndrome donors (METS-D) induced decrease of insulin sensitivity, post-Roux-en-Y gastric bypass donors (RYGB-D) could improve insulin sensitivity and altered expression of metabolism related molecules (de Groot et al., 2020; Kootte et al., 2017).

Interestingly, populations traditionally considered as recipients for FMT may also possess the potential to serve as donors under certain circumstances. Healthy overweight or obese donors have been included to treat cachectic patients with advanced gastroesophageal cancer, although this FMT process prior to first-line chemotherapy did not improve cachexia, improved response and survival in patients with metastatic gastroesophageal cancer was observed (de Clercq et al., 2021).

Meanwhile, at the level of recipients, researchers are exploring the extra measures that recipients could adopt post-FMT to maximize the benefits of FMT treatment. The development of specific lifestyle or diet post-FMT may serve as a promoter for the development and maintenance of the intestinal type, which is favorable to disease recovery. Oral pectin intake for five consecutive days following FMT enhances the effect of FMT in UC by maintaining gut microbial diversity, which could be fermented into SCFAs (Wei et al., 2016). FMT plus lifestyle intervention resulted in greater efficacy of microbiota engraftment from donors in recipients with type 2 diabetes post-FMT (Ng et al., 2022). Patients with mild to moderate UC who accepted FMT and anti-inflammatory diet showed more profound deep remission than those who received stable baseline medications (Kedia et al., 2022). FMT coupled with dietary fiber intervention contributes to shape gut microbiota composition and improve the effectiveness of FMT in recipients (Zhong et al., 2021). This finding was further validated in a randomized double-blind trail, revealing that during FMT, supplementation of low-fermentable fiber, but not high-fermentable fiber, led to better outcome of obesity and metabolic syndrome (Mocanu et al., 2021). Specially, a microbiome-based artificial intelligence-assisted personalized diet significantly reduced IBS-SSS scores across all IBS subtypes, whose efficacy was even better than low-fermentable diet, revealing the prospect of artificial intelligence-assisted therapy in the field of FMT (Tunali et al., 2024).

In short, in the future, screening for the metabolic status and shaping lifestyle habits in donors and recipients should be further performed, which may lead to more stable clinical remission without accompanying metabolic disorders. Furthermore, we should not overlook the potential beneficial effects that the feces of “sub-healthy” individuals with specific metabolic characteristics may have on recipients.

## 12 Bowel preparation and medication application in recipients

As previously mentioned, the efficacy of FMT largely depends on the interaction between the gut microbiota of the donor and the recipient. Therefore, in addition to donor screening based on microbial community characteristics, pre-regulating the gut microbiota of recipients may contribute to the success rate of FMT.

Researches showed bowel cleansing could enhance donor microbiome engraftment, inadequate bowel preparation is an independent predictor of failure after single FMT in treating rCDI (Ianiro et al., 2017; Podlesny et al., 2022). While in addition to the use of intestinal preparation drugs such as polyethylene glycol, according to the international guidelines of FMT, patients with rCDI should be treated with antibiotics including vancomycin or fidaxomicin at least for 3 days pre-FMT to lower the abundance of intestinal *C. difficile*, and antibiotics should be stopped 12 to 48 h pre-FMT (Cammarota et al., 2017). However, in diseases other than rCDI, no exact recommendation has been proposed.

Current research on the use of antibiotics before FMT is preparing the groundwork for the development of more refined guidelines in the future. The reconstruction of gut microbiome occurs against intestinal antibiotic exposure, while due to the complex ecological network of intestine, the effect of antibiotics varies in different individuals. Overall, in terms of multi-species population, administration of antibiotics could influence the following factors of microbiota within patients' gut: taxonomic and resistome composition, nutrient availability and complexity, metabolic networks of cross-feeding or competition, and horzontal gene transfer of resistome elements, shaping the recipients' gut microbiota to better introduce those of the donors (Fishbein et al., 2023). A metagenomic systematic meta-analysis of 24 studies showed that antibiotics used pre-FMT was positively-associated with donor strain engraftment and clinical success (Ianiro et al., 2022). The administration of antibiotics pre-FMT in recipients showed better modification of gut microbiota and increased xenomicrobiota colonization post-FMT, compared to performing bowel cleansing solution or no pretreatment (Ji et al., 2017). Species-level dysbiosis within the phylum *Bacteroidetes* could be found in the gut of UC patients, triple-antibiotic pre-treatment in these patients, including amoxicillin, fosfomycin and metronidazole, could promote the eradication of dysbiotic strains and further colonization of viable *Bacteroidetes* cells (Ishikawa et al., 2018). Another study also utilized a triple-antibiotic pre-treatment, including amoxicillin, doxycycline, and metronidazole, which also reached promising results (Haifer et al., 2022). The greater engraftment of strains in recipients provided by antibiotics used pre-FMT was also discovered in immunosuppressed patients, such as individuals with HIV (Serrano-Villar et al., 2021). In the safety study of SER-287, a spore-based microbiome therapeutic, in treating UC, the group vancomycin/SER-287 showed higher proportion of patients who achieved clinical remission than the placebo/SER-287 group (Henn et al., 2021).

Different therapeutic courses of certain antibiotics before FMT could result in distinct clinical outcomes post-FMT. An animal experiment proved that both frequency of dosing and duration of preparative antibiotic treatment influences strain engraftment post-FMT (Gopalakrishnan et al., 2021). In human, short duration of vancomycin pre-treatment is positively related with the positive C. difficile culture pre-FMT and early CDI recurrence (Groenewegen et al., 2024). In a retrospective study of rCDI treatment, oral vancomycin for standard duration of 10–14 days pre-FMT was not significantly associated with FMT success, while prolonged vancomycin taper for ≥6 weeks induced better outcome in FMT treatment. Mechanistically, standard therapeutic courses of vancomycin may reduce the stool concentrations of *C. difficile* and allow the persistence of disrupted microbiota. In contrast, the prolonged tapered course of oral vancomycin pre-FMT may result in partial microbiota replenishment and reducing the fecal concentration of vegetative C. difficile (Patron et al., 2017).

However, the application of antibiotics prior to FMT has, at times, been proved to be ineffective in enhancing treatment efficacy. In a randomized controlled trial, individuals with IBS-D received pre-FMT ciprofloxacin and metronidazole (CM-FMT) or rifaximin (R-FMT) conversely exhibited a lower rate of microbial strain engraftment compared to individuals with alone FMT (Singh et al., 2022). Moreover, paradoxically, the exposure to systemic antibiotic therapy might be related to the onset of dysbiosis-related chronic diseases including IBD (Nguyen et al., 2020). The elevated susceptibility to IBD is most frequently observed in population aged over 40. The highest risk of IBD onset is typically seen 1–2 years post-antibiotic administration and after the use of antibiotics commonly prescribed to treat gastrointestinal infections (Faye et al., 2023). Antibiotic exposure is also the highest risk for CDI (Pérez-Cobas et al., 2015). An animal experiment proved that short-term exposure to antibiotics could induce lowered microbial alpha and beta diversities, reduced complexity of gut molecular ecological networks, and impaired microbial metabolic pathways related with bacterial survival and physiological functions. These mentioned changes could not entirely recover over time. Although the antibiotic administration pre-FMT would reshape the impaired microecology to some degree, we still should not ignore the risk of long-term side effects induced by short-term antibiotics intervention (Hong et al., 2024). These findings remind us that further research is still warranted to confirm the benefits of antibiotic-based gut preparation pre-FMT. Caution must be exercised to regard the potential ineffectiveness of the treatment and the risk of illness aggravation antibiotics may pose.

Except for using antibiotics at the time of preparation before FMT, if antibiotics are administered at the wrong time point during the FMT process, they can also become ineffective or even become an obstacle to the effectiveness of the treatment. In the scope of pre-FMT, feces from human donors who administrated repeated, but not recent antibiotics might induce impaired mucus growth mucus barrier integrity in mice recipients, the microbiota community of which is characterized by enriched mucus-utilizing bacteria, including *Akkermansia muciniphila* and *Bacteroides fragilis*. As a result of altered microbiota composition, 10 metabolites, including adenine, adenosine and betaine, etc., showed significantly altered abundance in mice recipients (Krigul et al., 2024). While in the scope of post-FMT, non-CDI antibiotics during follow-up were all independently associated with a lower treatment effect from FMT in CDI treatment (Baunwall et al., 2023; Groenewegen et al., 2024). Antibiotic use within the first 8 weeks after FMT may disrupt microbial engraftment and lower the success rate of FMT (Allegretti et al., 2018).

In summary, the creation of a microenvironment conducive to microbial engraftment within the gut of recipients is of great importance pre-FMT, which could be achieving through the application of antibiotics pre-FMT. Specially, factors including the time point, duration, and type of antibiotics administration also influence clinical outcomes.

## 13 Conclusion and perspective

As a microbiome-based therapy, the primary change observed after FMT in recipients is the alteration of the gut microbiota. In this review, we summarized diseases that could be treated with FMT, which are characterized by microbial dysbiosis, with distinct baseline gut microbiome impairment, and the reshaping of the gut microbiota could be detected post-FMT. Some diseases have been widely recognized as targets for FMT treatment and are recommended in several conferences and guidelines, such as CDI. Some diseases still require further clinical trials to validate their application conditions and therapeutic value, such as IBD and IBS. Interestingly, some newly discovered potential diseases, which might have a response to FMT, are still in the initial stages of clinical trials or animal experiments. Therefore, FMT holds immense potential in the treatment of both gastrointestinal and extraintestinal diseases.

Furthermore, this article systematically summarized the critical factors affecting the efficacy of FMT at the donor and recipient levels. The interaction between donor and recipient microbiota is crucial in FMT, and our review outlined the fusion patterns of the gut microbiota post-FMT, which conduces to the understanding of different clinical outcomes. The interplay between the donr and the recipient is a complicated process, and researchers have developed various predictive models for FMT based on regression or machine learning methods, which have significant guidance value in clinical practice.

**Figure 2:**
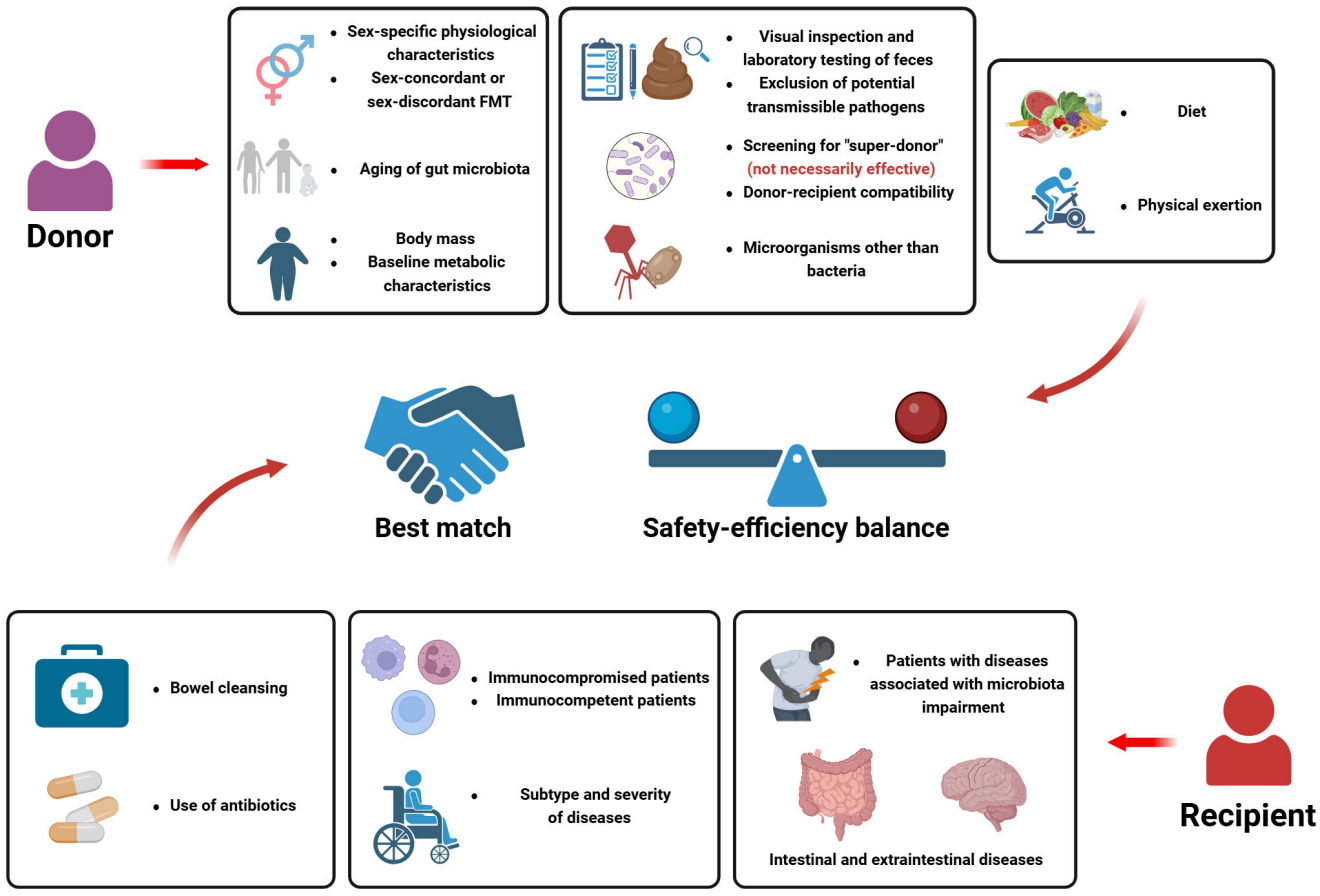
Potential factors influencing the efficacy of FMT from the perspective of donors and recipients.

In addition to gut microbiota-related factors, we have also listed other determinants of FMT safety and efficacy. Pre-testing of potential pathogens in donor feces is of great importance. There is also a need to assess the immune status of the recipient, although the recipient's immunocompromised state does not necessarily affect the conduct of FMT. The values of baseline characteristics of donors and recipients, including gender, age, clinical status, lifestyle and application of antibiotics, etc., have also been discussed in our review (Figure 2). The importance of these potential regulatory factors have been proved in clinical trails and animal experiments, while several researches have also provided opposite results.

In summary, this review comprehensively explored the application scenarios and conditions of FMT, expounded “what constitutes a successful FMT treatment”, and how the interplay between donors and recipients affects treatment outcomes. More research is still needed to further guide the application of FMT, to improve the current situation where FMT is regarded as a “black box” to a certain extent, to enhance the explainablity and transparency of FMT treatment and related predictive models, and to extend the application scope of FMT.

Key-points of this review:

(1) We provide a systematical summary of the recognized or potential diseases that can be treated by FMT, and the microbial and metabolic characteristics of the diseases pre- and post-FMT.

(2) We introduce the microbial donor-recipient interplay and the lasting microbial succession within the gut post-FMT, which is influenced by the baseline microbial characteristics of both the donor and the recipient.

(3) We summarize the prognostic models on FMT efficacy to guide clinical decision-making.

(4) We analyze the potential role of several confounding factors (e.g., immune system function, age, gender, lifestyle, antibiotic use, etc) in the safety and efficiency of FMT from the perspectives of both donors and recipients. In this field, we include some early-stage studies, which have not yet reached clear conclusions or have obtained contradictory results, laying a foundation for future research.

(5) Combining the key points mentioned above, we clarify how to conduct a successful FMT at appropriate application scenarios.

## References

[B1] Abenavoli L. Gambardella M. L. Scarlata G. Lenci I. Baiocchi L. Luzza F. (2024). The many faces of metabolic dysfunction-associated fatty liver disease treatment: from the mediterranean diet to fecal microbiota transplantation. Medicina 60:563. 10.3390/medicina60040563 38674209 PMC11051743

[B2] Afzaal M. Saeed F. Shah Y. A. Hussain M. Rabail R. Socol C. T.. (2022). Human gut microbiota in health and disease: unveiling the relationship. Front. Microbiol. 13:999001. 10.3389/fmicb.2022.999001 36225386 PMC9549250

[B3] Aggarwala V. Mogno I. Li Z. Yang C. Britton G. J. Chen-Liaw A.. (2021). Precise quantification of bacterial strains after fecal microbiota transplantation delineates long-term engraftment and explains outcomes. Nat. Microbiol. 6, 1309–1318. 10.1038/s41564-021-00966-0 34580445 PMC8993687

[B4] Allegretti J. R. Kao D. Sitko J. Fischer M. Kassam Z. (2018). Early antibiotic use after fecal microbiota transplantation increases risk of treatment failure. Clin. Infect. Dis. 66, 134–135. 10.1093/cid/cix684 29020157

[B5] Anand R. Song Y. Garg S. Girotra M. Sinha A. Sivaraman A.. (2017). Effect of aging on the composition of fecal microbiota in donors for FMT and its impact on clinical outcomes. Dig. Dis. Sci. 62, 1002–1008. 10.1007/s10620-017-4449-6 28181098

[B6] Andersen V. Pingel J. Søfelt H. L. Hikmat Z. Johansson M. Pedersen V. S.. (2024). Sex and gender in inflammatory bowel disease outcomes and research. Lancet Gastroenterol. Hepatol. 9, 1041–1051. 10.1016/S2468-1253(24)00159-6 39395438

[B7] Arzamendi M. J. Habibyan Y. B. Defaye M. Shute A. Baggio C. H. Chan R.. (2024). Sex-specific post-inflammatory dysbiosis mediates chronic visceral pain in colitis. Gut Microbes 16, 2409207. 10.1080/19490976.2024.2409207 39360560 PMC11451282

[B8] Barandouzi Z. A. Starkweather A. R. Henderson W. A. Gyamfi A. Cong X. S. (2020). Altered composition of gut microbiota in depression: a systematic review. Front. Psychiatry 11:541. 10.3389/fpsyt.2020.00541 32587537 PMC7299157

[B9] Baunwall S. Hansen M. M. Andreasen S. E. Eriksen M. K. Rågård N. Kelsen J.. (2023). Donor, patient age and exposure to antibiotics are associated with the outcome of faecal microbiota transplantation for recurrent *Clostridioides difficile* infection: a prospective cohort study. Aliment. Pharmacol. Ther. 58, 503–515. 10.1111/apt.17642 37482926

[B10] Behling A. H. Wilson B. C. Ho D. Cutfield W. S. Vatanen T. O'Sullivan. (2024). Horizontal gene transfer after faecal microbiota transplantation in adolescents with obesity. Microbiome 12:26. 10.1186/s40168-024-01748-6 38347627 PMC10860221

[B11] Benech N. Cassir N. Galperine T. Alric L. Scanzi J. Sokol H. (2024). Fecal microbiota transplantation for recurrent *Clostridioides difficile* infection can be the best therapeutic option in severely immunocompromised patients depending on a case-by-case assessment of the benefit-to-risk ratio. Gastroenterology 167, 627–628. 10.1053/j.gastro.2024.04.022 38679396

[B12] Benítez-Páez A. Hartstra A. V. Nieuwdorp M. Sanz Y. (2022). Species- and strain-level assessment using rrn long-amplicons suggests donor's influence on gut microbial transference via fecal transplants in metabolic syndrome subjects. Gut Microbes 14:2078621. 10.1080/19490976.2022.2078621 35604764 PMC9132484

[B13] Beran A. Sharma S. Ghazaleh S. Lee-Smith W. Aziz M. Kamal F.. (2023). Predictors of fecal microbiota transplant failure in *Clostridioides difficile* infection: an updated meta-analysis. J. Clin. Gastroenterol. 57, 389–399. 10.1097/MCG.0000000000001667 35050941

[B14] Berer K. Gerdes L. A. Cekanaviciute E. Jia X. Xiao L. Xia Z.. (2017). Gut microbiota from multiple sclerosis patients enables spontaneous autoimmune encephalomyelitis in mice. Proc. Natl. Acad. Sci. U.S.A. 114, 10719–10724. 10.1073/pnas.1711233114 28893994 PMC5635914

[B15] Browne P. D. Cold F. Petersen A. M. Halkjær S. I. Christensen A. H. Günther S.. (2021). Engraftment of strictly anaerobic oxygen-sensitive bacteria in irritable bowel syndrome patients following fecal microbiota transplantation does not improve symptoms. Gut Microbes 13, 1–16. 10.1080/19490976.2021.1927635 34074214 PMC8183560

[B16] Bruggeman A. Vandendriessche C. Hamerlinck H. De Looze D. Tate D. J. Vuylsteke M.. (2024). Safety and efficacy of faecal microbiota transplantation in patients with mild to moderate Parkinson's disease (GUT-PARFECT): a double-blind, placebo-controlled, randomised, phase 2 trial. EClinicalMedicine 71:102563. 10.1016/j.eclinm.2024.102563 38686220 PMC11056595

[B17] Caenepeel C. Deleu S. Vazquez Castellanos J. F. Arnauts K. Braekeleire S. Machiels K.. (2024). Rigorous donor selection for fecal microbiota transplantation in active ulcerative colitis: key lessons from a randomized controlled trial halted for futility. Clin. Gastroenterol. Hepatol. 23, 621–631.e7. 10.1016/j.cgh.2024.05.017 38788915

[B18] Cammarota G. Ianiro G. Tilg H. Rajilić-Stojanovi,ć M. Kump P. Satokari R.. (2017). European consensus conference on faecal microbiota transplantation in clinical practice. Gut 66, 569–580. 10.1136/gutjnl-2016-313017 28087657 PMC5529972

[B19] Chen Q. Fan Y. Zhang B. Yan C. Chen Z. Wang L.. (2022). Specific fungi associated with response to capsulized fecal microbiota transplantation in patients with active ulcerative colitis. Front. Cell. Infect. Microbiol. 12:1086885. 10.3389/fcimb.2022.1086885 36683707 PMC9849685

[B20] Chen Q. Wu C. Xu J. Ye C. Chen X. Tian H.. (2024). Donor-recipient intermicrobial interactions impact transfer of subspecies and fecal microbiota transplantation outcome. Cell Host Microbe 32, 349–365.e4. 10.1016/j.chom.2024.01.013 38367621

[B21] Cheng C. K. Gao J. Kang L. Huang Y. (2024). Fecal microbiota transfer from young mice reverts vascular aging hallmarks and metabolic impairments in aged mice. Aging Dis. 10.14336/AD.2024.0384 39012675 PMC12096931

[B22] Cheng Y. W. Phelps E. Ganapini V. Khan N. Ouyang F. Xu H.. (2019). Fecal microbiota transplantation for the treatment of recurrent and severe *Clostridium difficile* infection in solid organ transplant recipients: a multicenter experience. Am. J. Transplant. 19, 501–511. 10.1111/ajt.15058 30085388 PMC6349556

[B23] Chen-Liaw A. Aggarwala V. Mogno I. Haifer C. Li Z. Eggers J.. (2024). Gut microbiota strain richness is species specific and affects engraftment. Nature 637, 422–429. 10.1038/s41586-024-08242-x 39604726 PMC12547691

[B24] Chuang C. Lee K. C. Wang Y. P. Lee P. C. Chang T. E. Huang Y. H.. (2023). High carriage rate of extended-spectrum β-lactamase Enterobacterales and diarrheagenic *Escherichia coli* in healthy donor screening for fecal microbiota transplantation. Eur. J. Clin. Microbiol. Infect. Dis. 42, 1103–1113. 10.1007/s10096-023-04644-3 37474764

[B25] Conover K. R. Absah I. Ballal S. Brumbaugh D. Cho S. Cardenas M. C.. (2023). Fecal microbiota transplantation for *Clostridioides difficile* infection in immunocompromised pediatric patients. J. Pediatr. Gastroenterol. Nutr. 76, 440–446. 10.1097/MPG.0000000000003714 36720105 PMC10627107

[B26] Costello S. P. Soo W. Bryant R. V. Jairath V. Hart A. L. Andrews J. M. (2017). Systematic review with meta-analysis: faecal microbiota transplantation for the induction of remission for active ulcerative colitis. Aliment. Pharmacol. Ther. 46, 213–224. 10.1111/apt.14173 28612983

[B27] Cui M. Xiao H. Li Y. Zhou L. Zhao S. Luo D.. (2017). Faecal microbiota transplantation protects against radiation-induced toxicity. EMBO Mol. Med. 9, 448–461. 10.15252/emmm.201606932 28242755 PMC5376756

[B28] D'Amato A. Di Cesare Mannelli L. Lucarini E. Man A. L. Le Gall G.. (2020). Faecal microbiota transplant from aged donor mice affects spatial learning and memory via modulating hippocampal synaptic plasticity- and neurotransmission-related proteins in young recipients. Microbiome 8:140. 10.1186/s40168-020-00914-w 33004079 PMC7532115

[B29] de Clercq N. C. van den Ende T. Prodan A. Hemke R. Davids M. Pedersen H. K.. (2021). Fecal microbiota transplantation from overweight or obese donors in cachectic patients with advanced gastroesophageal cancer: a randomized, double-blind, placebo-controlled, phase II study. Clin. Cancer Res. 27, 3784–3792. 10.1158/1078-0432.CCR-20-4918 33883174

[B30] de Groot P. Nikolic T. Pellegrini S. Sordi V. Imangaliyev S. Rampanelli E.. (2021). Faecal microbiota transplantation halts progression of human new-onset type 1 diabetes in a randomised controlled trial. Gut 70, 92–105. 10.1136/gutjnl-2020-322630 33106354 PMC7788262

[B31] de Groot P. Scheithauer T. Bakker G. J. Prodan A. Levin E. Khan M. T.. (2020). Donor metabolic characteristics drive effects of faecal microbiota transplantation on recipient insulin sensitivity, energy expenditure and intestinal transit time. Gut 69, 502–512. 10.1136/gutjnl-2019-318320 31147381 PMC7034343

[B32] DeFilipp Z. Bloom P. P. Torres Soto M. Mansour M. K. Sater M. Huntley M. H.. (2019). Drug-resistant *E. coli* bacteremia transmitted by fecal microbiota transplant. N. Engl. J. Med. 381, 2043–2050. 10.1056/NEJMoa1910437 31665575

[B33] Drewes J. L. Corona A. Sanchez U. Fan Y. Hourigan S. K. Weidner M.. (2019). Transmission and clearance of potential procarcinogenic bacteria during fecal microbiota transplantation for recurrent *Clostridioides difficile*. JCI Insight 4:130848. 10.1172/jci.insight.130848 31578306 PMC6795395

[B34] El-Salhy M. Hatlebakk J. G. Gilja O. H. Bråthen Kristoffersen A. Hausken T. (2020). Efficacy of faecal microbiota transplantation for patients with irritable bowel syndrome in a randomised, double-blind, placebo-controlled study. Gut 69, 859–867. 10.1136/gutjnl-2019-319630 31852769 PMC7229896

[B35] El-Salhy M. Valeur J. Hausken T. Gunnar Hatlebakk J. (2021). Changes in fecal short-chain fatty acids following fecal microbiota transplantation in patients with irritable bowel syndrome. Neurogastroenterol. Motil. 33:e13983. 10.1111/nmo.13983 32945066 PMC7900992

[B36] Faye A. S. Allin K. H. Iversen A. T. Agrawal M. Faith J. Colombel J. F.. (2023). Antibiotic use as a risk factor for inflammatory bowel disease across the ages: a population-based cohort study. Gut 72, 663–670. 10.1136/gutjnl-2022-327845 36623926 PMC9998355

[B37] Feješ A. Belvončíková P. Porcel Sanchis D. Borbélyová V. Celec P. DŽunková M.. (2024). The effect of cross-sex fecal microbiota transplantation on metabolism and hormonal status in adult rats. Int. J. Mol. Sci. 25:601. 10.3390/ijms25010601 38203771 PMC10778742

[B38] Fischer M. Kao D. Mehta S. R. Martin T. Dimitry J. Keshteli A. H.. (2016). Predictors of early failure after fecal microbiota transplantation for the therapy of *Clostridium difficile* infection: a multicenter study. Am. J. Gastroenterol. 111, 1024–1031. 10.1038/ajg.2016.180 27185076

[B39] Fishbein S. Mahmud B. Dantas G. (2023). Antibiotic perturbations to the gut microbiome. Nat. Rev. Microbiol. 21, 772–788. 10.1038/s41579-023-00933-y 37491458 PMC12087466

[B40] Fu Z. P. Ying Y. G. Wang R. Y. Wang Y. Q. (2024). Aged gut microbiota promotes arrhythmia susceptibility via oxidative stress. iScience 27:110888. 10.1016/j.isci.2024.110888 39381749 PMC11460473

[B41] Fuentes S. Rossen N. G. van der Spek M. J. Hartman J. H. Huuskonen L. Korpela K.. (2017). Microbial shifts and signatures of long-term remission in ulcerative colitis after faecal microbiota transplantation. ISME J. 11, 1877–1889. 10.1038/ismej.2017.44 28398347 PMC5520032

[B42] Fujimoto K. Kimura Y. Allegretti J. R. Yamamoto M. Zhang Y. Z. Katayama K.. (2021). Functional restoration of bacteriomes and viromes by fecal microbiota transplantation. Gastroenterology 160, 2089–2102.e12. 10.1053/j.gastro.2021.02.013 33577875 PMC8684800

[B43] Gallo A. Cancelli C. Ceron E. Covino M. Capoluongo E. Pocino K.. (2020). Fecal calprotectin and need of multiple microbiota trasplantation infusions in *Clostridium difficile* infection. J. Gastroenterol. Hepatol. 35, 1909–1915. 10.1111/jgh.15072 32291810

[B44] Ge C. Lu Y. Shen Z. Lu Y. Liu X. Zhang M.. (2025). Machine learning and metabolomics identify biomarkers associated with the disease extent of ulcerative colitis. J. Crohn's Colitis 19:jjaf020. 10.1093/ecco-jcc/jjaf020 39903649 PMC11829215

[B45] Ghani R. Chrysostomou D. Roberts L. A. Pandiaraja M. Marchesi J. R. Mullish B. H. (2024). Faecal (or intestinal) microbiota transplant: a tool for repairing the gut microbiome. Gut Microbes 16:2423026. 10.1080/19490976.2024.2423026 39499189 PMC11540080

[B46] Gogokhia L. Buhrke K. Bell R. Hoffman B. Brown D. G. Hanke-Gogokhia C.. (2019). Expansion of bacteriophages is linked to aggravated intestinal inflammation and colitis. Cell Host Microbe 25, 285–299.e8. 10.1016/j.chom.2019.01.008 30763538 PMC6885004

[B47] Goldsmith J. Tomkovich S. Auninš J. G. McGovern B. H. Mahoney J. C. Hasson B. R.. (2024). End-to-end donor screening and manufacturing controls: complementary quality-based strategies to minimize patient risk for donor-derived microbiome therapeutics. Gut Microbes 16:2402550. 10.1080/19490976.2024.2402550 39292598 PMC11529408

[B48] Gopalakrishnan V. Dozier E. A. Glover M. S. Novick S. Ford M. Morehouse C.. (2021). Engraftment of bacteria after fecal microbiota transplantation is dependent on both frequency of dosing and duration of preparative antibiotic regimen. Microorganisms 9:1399. 10.3390/microorganisms9071399 34209573 PMC8306289

[B49] Gough E. Shaikh H. Manges A. R. (2011). Systematic review of intestinal microbiota transplantation (fecal bacteriotherapy) for recurrent *Clostridium difficile* infection. Clin. Infect. Dis. 53, 994–1002. 10.1093/cid/cir632 22002980

[B50] Groenewegen B. van Lingen E. Kovynev A. van den Berg A. J. Berssenbrugge E. Sanders I.. (2024). The presence of *Clostridioides difficile* in faeces before and after faecal microbiota transplantation and its relation with recurrent C. difficile infection and the gut microbiota in a Dutch cohort. Clin. Microbiol. Infect. 10.1016/j.cmi.2024.12.003 39662821

[B51] Gupta A. Khanna S. (2017). Fecal microbiota transplantation. JAMA 318:102. 10.1001/jama.2017.6466 28672320

[B52] Gupta S. Allen-Vercoe E. Petrof E. O. (2016). Fecal microbiota transplantation: in perspective. Therap. Adv. Gastroenterol. 9, 229–239. 10.1177/1756283X15607414 26929784 PMC4749851

[B53] Haifer C. Paramsothy S. Kaakoush N. O. Saikal A. Ghaly S. Yang T.. (2022). Lyophilised oral faecal microbiota transplantation for ulcerative colitis (LOTUS): a randomised, double-blind, placebo-controlled trial. Lancet Gastroenterol. Hepatol. 7, 141–151. 10.1016/S2468-1253(21)00400-3 34863330

[B54] Halkjær S. I. Christensen A. H. Lo B. Browne P. D. Günther S. Hansen L. H.. (2018). Faecal microbiota transplantation alters gut microbiota in patients with irritable bowel syndrome: results from a randomised, double-blind placebo-controlled study. Gut 67, 2107–2115. 10.1136/gutjnl-2018-316434 29980607

[B55] Han L. Zhao L. Zhou Y. Yang C. Xiong T. Lu L.. (2022). Altered metabolome and microbiome features provide clues in understanding irritable bowel syndrome and depression comorbidity. ISME J. 16, 983–996. 10.1038/s41396-021-01123-5 34750528 PMC8940891

[B56] Haro C. Rangel-Zúñiga O. A. Alcalá-Díaz J. F. Gómez-Delgado F. Pérez-Martínez P. Delgado-Lista J.. (2016). Intestinal microbiota is influenced by gender and body mass index. PLoS ONE 11:e0154090. 10.1371/journal.pone.0154090 27228093 PMC4881937

[B57] He R. Li P. Wang J. Cui B. Zhang F. Zhao F. (2022). The interplay of gut microbiota between donors and recipients determines the efficacy of fecal microbiota transplantation. Gut Microbes 14:2100197. 10.1080/19490976.2022.2100197 35854629 PMC9302524

[B58] Henn M. R. O'Brien E. J. Diao L. Feagan B. G. Sandborn W. J.. (2021). A phase 1b safety study of SER-287, a spore-based microbiome therapeutic, for active mild to moderate ulcerative colitis. Gastroenterology 160, 115–127.e30. 10.1053/j.gastro.2020.07.048 32763240 PMC7402096

[B59] Hocquart M. Lagier J. C. Cassir N. Saidani N. Eldin C. Kerbaj J.. (2018). Early fecal microbiota transplantation improves survival in severe *Clostridium difficile* infections. Clin. Infect. Dis. 66, 645–650. 10.1093/cid/cix762 29020328

[B60] Hong Y. Li H. Chen L. Su H. Zhang B. Luo Y.. (2024). Short-term exposure to antibiotics begets long-term disturbance in gut microbial metabolism and molecular ecological networks. Microbiome 12:80. 10.1186/s40168-024-01795-z 38715137 PMC11075301

[B61] Hourigan S. K. Nicholson M. R. Kahn S. A. Kellermayer R. (2021). Updates and challenges in fecal microbiota transplantation for *Clostridioides difficile* infection in children. J. Pediatr. Gastroenterol. Nutr. 73, 430–432. 10.1097/MPG.0000000000003229 34238831 PMC8455422

[B62] Huang C. Yi P. Zhu M. Zhou W. Zhang B. Yi X.. (2022). Safety and efficacy of fecal microbiota transplantation for treatment of systemic lupus erythematosus: an EXPLORER trial. J. Autoimmun. 130:102844. 10.1016/j.jaut.2022.102844 35690527

[B63] Ianiro G. Mullish B. H. Kelly C. R. Kassam Z. Kuijper E. J. Ng S. C.. (2020). Reorganisation of faecal microbiota transplant services during the COVID-19 pandemic. Gut 69, 1555–1563. 10.1136/gutjnl-2020-321829 32620549 PMC7456726

[B64] Ianiro G. Punčochár M. Karcher N. Porcari S. Armanini F. Asnicar F.. (2022). Variability of strain engraftment and predictability of microbiome composition after fecal microbiota transplantation across different diseases. Nat. Med. 28, 1913–1923. 10.1038/s41591-022-01964-3 36109637 PMC9499858

[B65] Ianiro G. Valerio L. Masucci L. Pecere S. Bibb,ò S. Quaranta G.. (2017). Predictors of failure after single faecal microbiota transplantation in patients with recurrent *Clostridium difficile* infection: results from a 3-year, single-centre cohort study. Clin. Microbiol. Infect. 23, 337.e1–337.e3. 10.1016/j.cmi.2016.12.025 28057560

[B66] Ishikawa D. Sasaki T. Takahashi M. Kuwahara-Arai K. Haga K. Ito S.. (2018). The microbial composition of bacteroidetes species in ulcerative colitis is effectively improved by combination therapy with fecal microbiota transplantation and antibiotics. Inflamm. Bowel Dis. 24, 2590–2598. 10.1093/ibd/izy266 30124831

[B67] Jacobs J. P. Lagishetty V. Hauer M. C. Labus J. S. Dong T. S. Toma R.. (2023). Multi-omics profiles of the intestinal microbiome in irritable bowel syndrome and its bowel habit subtypes. Microbiome 11:5. 10.1186/s40168-022-01450-5 36624530 PMC9830758

[B68] Ji S. K. Yan H. Jiang T. Guo C. Y. Liu J. J. Dong S. Z.. (2017). Preparing the gut with antibiotics enhances gut microbiota reprogramming efficiency by promoting xenomicrobiota colonization. Front. Microbiol. 8:1208. 10.3389/fmicb.2017.01208 28702022 PMC5487471

[B69] Johnsen P. H. Hilpüsch F. Cavanagh J. P. Leikanger I. S. Kolstad C. Valle P. C.. (2018). Faecal microbiota transplantation versus placebo for moderate-to-severe irritable bowel syndrome: a double-blind, randomised, placebo-controlled, parallel-group, single-centre trial. Lancet Gastroenterol. Hepatol. 3, 17–24. 10.1016/S2468-1253(17)30338-2 29100842

[B70] Kang G. U. Park S. Jung Y. Jee J. J. Kim M. S. Lee S.. (2022). Exploration of potential gut microbiota-derived biomarkers to predict the success of fecal microbiota transplantation in ulcerative colitis: a prospective cohort in Korea. Gut Liver 16, 775–785. 10.5009/gnl210369 35975640 PMC9474483

[B71] Karimi M. Shirsalimi N. Hashempour Z. Salehi Omran H. Sedighi E. Beigi F.. (2024). Safety and efficacy of fecal microbiota transplantation (FMT) as a modern adjuvant therapy in various diseases and disorders: a comprehensive literature review. Front. Immunol. 15, 1439176. 10.3389/fimmu.2024.1439176 39391303 PMC11464302

[B72] Kazemian N. Ramezankhani M. Sehgal A. Khalid F. M. Kalkhoran A. Narayan A.. (2020). The trans-kingdom battle between donor and recipient gut microbiome influences fecal microbiota transplantation outcome. Sci. Rep. 10:18349. 10.1038/s41598-020-75162-x 33110112 PMC7591866

[B73] Kedia S. Virmani S. K Vuyyuru S. Kumar P. Kante B.. (2022). Faecal microbiota transplantation with anti-inflammatory diet (FMT-AID) followed by anti-inflammatory diet alone is effective in inducing and maintaining remission over 1 year in mild to moderate ulcerative colitis: a randomised controlled trial. Gut 71, 2401–2413. 10.1136/gutjnl-2022-327811 35973787

[B74] Keskey R. Cone J. T. DeFazio J. R. Alverdy J. C. (2020). The use of fecal microbiota transplant in sepsis. Transl. Res. 226, 12–25. 10.1016/j.trsl.2020.07.002 32649987 PMC7572598

[B75] Khoruts A. (2021). Can FMT cause or prevent CRC? Maybe, but there is more to consider. Gastroenterology 161, 1103–1105. 10.1053/j.gastro.2021.06.074 34224741

[B76] Koo H. Morrow C. D. (2021). Incongruence between dominant commensal donor microbes in recipient feces post fecal transplant and response to anti-PD-1 immunotherapy. BMC Microbiol. 21:251. 10.1186/s12866-021-02312-0 34544375 PMC8454007

[B77] Koo H. Morrow C. D. (2022). Time series strain tracking analysis post fecal transplantation identifies individual specific patterns of fecal dominant donor, recipient, and unrelated microbial strains. PLoS ONE 17:e0274633. 10.1371/journal.pone.0274633 36107983 PMC9477264

[B78] Kootte R. S. Levin E. Salojärvi J. Smits L. P. Hartstra A. V. Udayappan S. D.. (2017). Improvement of insulin sensitivity after lean donor feces in metabolic syndrome is driven by baseline intestinal microbiota composition. Cell Metab. 26, 611–619.e6. 10.1016/j.cmet.2017.09.008 28978426

[B79] Kragsnaes M. S. Kjeldsen J. Horn H. C. Munk H. L. Pedersen J. K. Just S. A.. (2021). Safety and efficacy of faecal microbiota transplantation for active peripheral psoriatic arthritis: an exploratory randomised placebo-controlled trial. Ann. Rheum. Dis. 80, 1158–1167. 10.1136/annrheumdis-2020-219511 33926922

[B80] Krigul K. L. Feeney R. H. Wongkuna S. Aasmets O. Holmberg S. M. Andreson R.. (2024). A history of repeated antibiotic usage leads to microbiota-dependent mucus defects. Gut Microbes 16:2377570. 10.1080/19490976.2024.2377570 39034613 PMC11529412

[B81] Kump P. Wurm P. Gröchenig H. P. Wenzl H. Petritsch W. Halwachs B.. (2018). The taxonomic composition of the donor intestinal microbiota is a major factor influencing the efficacy of faecal microbiota transplantation in therapy refractory ulcerative colitis. Aliment. Pharmacol. Ther. 47, 67–77. 10.1111/apt.14387 29052237 PMC5765501

[B82] Lacy B. E. Patel N. K. (2017). Rome Criteria and a Diagnostic Approach to Irritable Bowel Syndrome. J. Clin. Med. 6:99. 10.3390/jcm6110099 29072609 PMC5704116

[B83] Lee M. Chang E. B. (2021). Inflammatory bowel diseases (IBD) and the microbiome-searching the crime scene for clues. Gastroenterology 160, 524–537. 10.1053/j.gastro.2020.09.056 33253681 PMC8098834

[B84] Leonardi I. Paramsothy S. Doron I. Semon A. Kaakoush N. O. Clemente J. C.. (2020). Fungal trans-kingdom dynamics linked to responsiveness to fecal microbiota transplantation (FMT) therapy in ulcerative colitis. Cell Host Microbe 27, 823–829.e3. 10.1016/j.chom.2020.03.006 32298656 PMC8647676

[B85] Levy E. I. Dinleyici M. Dinleyici E. Vandenplas Y. (2024). *Clostridioides difficile* infections: prevention and treatment strategies. Adv. Exp. Med. Biol. 1449, 175–186. 10.1007/978-3-031-58572-2_11 39060738

[B86] Li N. Chen H. Cheng Y. Xu F. Ruan G. Ying S.. (2021). Fecal microbiota transplantation relieves gastrointestinal and autism symptoms by improving the gut microbiota in an open-label study. Front. Cell. Infect. Microbiol. 11:759435. 10.3389/fcimb.2021.759435 34737978 PMC8560686

[B87] Li S. S. Zhu A. Benes V. Costea P. I. Hercog R. Hildebrand F.. (2016). Durable coexistence of donor and recipient strains after fecal microbiota transplantation. Science 352, 586–589. 10.1126/science.aad8852 27126044

[B88] Liguori G. Lamas B. Richard M. L. Brandi G. da Costa G. Hoffmann T. W.. (2016). Fungal dysbiosis in mucosa-associated microbiota of Crohn's disease patients. J. Crohn's Colitis 10, 296–305. 10.1093/ecco-jcc/jjv209 26574491 PMC4957473

[B89] Liu Q. Zuo T. Lu W. Yeoh Y. K. Su Q. Xu Z.. (2022). Longitudinal evaluation of gut bacteriomes and viromes after fecal microbiota transplantation for eradication of carbapenem-resistant enterobacteriaceae. mSystems 7:e0151021. 10.1128/msystems.01510-21 35642928 PMC9239097

[B90] Lloyd-Price J. Arze C. Ananthakrishnan A. N. Schirmer M. Avila-Pacheco J. Poon T. W.. (2019). Multi-omics of the gut microbial ecosystem in inflammatory bowel diseases. Nature 569, 655–662. 10.1038/s41586-019-1237-9 31142855 PMC6650278

[B91] Lopetuso L. R. Deleu S. Godny L. Petito V. Puca P. Facciotti F.. (2023). The first international Rome consensus conference on gut microbiota and faecal microbiota transplantation in inflammatory bowel disease. Gut 72, 1642–1650. 10.1136/gutjnl-2023-329948 37339849 PMC10423477

[B92] MacLellan A. D. Finlay B. B. Appel-Cresswell S. (2021). Age-matching in pediatric fecal matter transplants. Front. Pediatr. 9:603423. 10.3389/fped.2021.603423 34336729 PMC8322514

[B93] Manrique P. Zhu Y. van der Oost J. Herrema H. Nieuwdorp M. de Vos W. M.. (2021). Gut bacteriophage dynamics during fecal microbial transplantation in subjects with metabolic syndrome. Gut Microbes 13, 1–15. 10.1080/19490976.2021.1897217 33794724 PMC8023239

[B94] Mao X. Larsen S. B. Zachariassen L. Brunse A. Adamberg S. Mejia J.. (2024). Transfer of modified gut viromes improves symptoms associated with metabolic syndrome in obese male mice. Nat. Commun. 15:4704. 10.1038/s41467-024-49152-w 38830845 PMC11148109

[B95] Marcella C. Cui B. Kelly C. R. Ianiro G. Cammarota G. Zhang F. (2021). Systematic review: the global incidence of faecal microbiota transplantation-related adverse events from 2000 to 2020. Aliment. Pharmacol. Ther. 53, 33–42. 10.1111/apt.16148 33159374

[B96] Mars R. Yang Y. Ward T. Houtti M. Priya S. Lekatz H. R.. (2020). Longitudinal multi-omics reveals subset-specific mechanisms underlying irritable bowel syndrome. Cell 182, 1460–1473.e17. 10.1016/j.cell.2020.08.007 32916129 PMC8109273

[B97] Martinez E. Taminiau B. Rodriguez C. Daube G. (2022). Gut microbiota composition associated with *Clostridioides difficile* colonization and infection. Pathogens 11:781. 10.3390/pathogens11070781 35890026 PMC9322938

[B98] Mehta N. Wang T. Friedman-Moraco R. J. Carpentieri C. Mehta A. K. Rouphael N.. (2022). Fecal microbiota transplantation donor screening updates and research gaps for solid organ transplant recipients. J. Clin. Microbiol. 60:e0016121. 10.1128/JCM.00161-21 34133889 PMC8849208

[B99] Meslier V. Laiola M. Roager H. M. De Filippis F. Roume H. Quinquis B.. (2020). Mediterranean diet intervention in overweight and obese subjects lowers plasma cholesterol and causes changes in the gut microbiome and metabolome independently of energy intake. Gut 69, 1258–1268. 10.1136/gutjnl-2019-320438 32075887 PMC7306983

[B100] Millan B. Park H. Hotte N. Mathieu O. Burguiere P. Tompkins T. A.. (2016). Fecal Microbial transplants reduce antibiotic-resistant genes in patients with recurrent *Clostridium difficile* infection. Clin. Infect. Dis. 62, 1479–1486. 10.1093/cid/ciw185 27025836 PMC4885654

[B101] Mills R. H. Dulai P. S. Vázquez-Baeza Y. Sauceda C. Daniel N. Gerner R. R.. (2022). Multi-omics analyses of the ulcerative colitis gut microbiome link *Bacteroides vulgatus* proteases with disease severity. Nat Microbiol 7, 262–276. 10.1038/s41564-021-01050-3 35087228 PMC8852248

[B102] Minkoff N. Z. Aslam S. Medina M. Tanner-Smith E. E. Zackular J. P. Acra S.. (2023). Fecal microbiota transplantation for the treatment of recurrent *Clostridioides difficile* (*Clostridium difficile*). Cochrane Database Syst. Rev. 4:CD013871. 10.1002/14651858.CD013871.pub2 37096495 PMC10125800

[B103] Mishra S. P. Wang B. Jain S. Ding J. Rejeski J. Furdui C. M.. (2023). A mechanism by which gut microbiota elevates permeability and inflammation in obese/diabetic mice and human gut. Gut 72, 1848–1865. 10.1136/gutjnl-2022-327365 36948576 PMC10512000

[B104] Moayyedi P. Surette M. G. Kim P. T. Libertucci J. Wolfe M. Onischi C.. (2015). Fecal microbiota transplantation induces remission in patients with active ulcerative colitis in a randomized controlled trial. Gastroenterology 149, 102–109.e6. 10.1053/j.gastro.2015.04.001 25857665

[B105] Mocanu V. Zhang Z. Deehan E. C. Kao D. H. Hotte N. Karmali S.. (2021). Fecal microbial transplantation and fiber supplementation in patients with severe obesity and metabolic syndrome: a randomized double-blind, placebo-controlled phase 2 trial. Nat. Med. 27, 1272–1279. 10.1038/s41591-021-01399-2 34226737

[B106] Mullish B. H. Merrick B. Quraishi M. N. Bak A. Green C. A. Moore D. J.. (2024). The use of faecal microbiota transplant as treatment for recurrent or refractory *Clostridioides difficile* infection and other potential indications: second edition of joint British Society of Gastroenterology (BSG) and Healthcare Infection Society (HIS) guidelines. J. Hosp. Infect. 148, 189–219. 10.1016/j.jhin.2024.03.001 38609760

[B107] Napiórkowska-Baran K. Biliński J. Pujanek M. Hałakuc P. Pietryga A. Szymczak B.. (2024). Fecal microbiota transplantation in a patient with chronic diarrhea and primary and secondary immunodeficiency (common variable immunodeficiency and splenectomy). Front. Cell. Infect. Microbiol. 14:1456672. 10.3389/fcimb.2024.1456672 39403201 PMC11472351

[B108] Newman T. M. Shively C. A. Register T. C. Appt S. E. Yadav H. Colwell R. R.. (2021). Diet, obesity, and the gut microbiome as determinants modulating metabolic outcomes in a non-human primate model. Microbiome 9:100. 10.1186/s40168-021-01069-y 33952353 PMC8101030

[B109] Ng S. C. Xu Z. Mak J. Yang K. Liu Q. Zuo T.. (2022). Microbiota engraftment after faecal microbiota transplantation in obese subjects with type 2 diabetes: a 24-week, double-blind, randomised controlled trial. Gut 71, 716–723. 10.1136/gutjnl-2020-323617 33785557

[B110] Nguyen L. H. Örtqvist A. K. Cao Y. Simon T. G. Roelstraete B. Song M.. (2020). Antibiotic use and the development of inflammatory bowel disease: a national case-control study in Sweden. Lancet Gastroenterol. Hepatol. 5, 986–995. 10.1016/S2468-1253(20)30267-3 32818437 PMC8034612

[B111] Ning L. Zhou Y. L. Sun H. Zhang Y. Shen C. Wang Z.. (2023). Microbiome and metabolome features in inflammatory bowel disease via multi-omics integration analyses across cohorts. Nat. Commun. 14:7135. 10.1038/s41467-023-42788-0 37932270 PMC10628233

[B112] Nishida A. Imaeda H. Ohno M. Inatomi O. Bamba S. Sugimoto M.. (2017). Efficacy and safety of single fecal microbiota transplantation for Japanese patients with mild to moderately active ulcerative colitis. J. Gastroenterol. 52, 476–482. 10.1007/s00535-016-1271-4 27730312

[B113] Nooij S. Ducarmon Q. R. Laros J. Zwittink R. D. Norman J. M. Smits W. K.. (2021). Fecal microbiota transplantation influences procarcinogenic escherichia coli in recipient recurrent *Clostridioides difficile* patients. Gastroenterology 161, 1218–1228.e5. 10.1053/j.gastro.2021.06.009 34126062

[B114] Nusbaum D. J. Sun F. Ren J. Zhu Z. Ramsy N. Pervolarakis N.. (2018). Gut microbial and metabolomic profiles after fecal microbiota transplantation in pediatric ulcerative colitis patients. FEMS Microbiol. Ecol. 94:fiy133. 10.1093/femsec/fiy133 30010747 PMC6454419

[B115] Paramsothy S. Nielsen S. Kamm M. A. Deshpande N. P. Faith J. J. Clemente J. C.. (2019). Specific bacteria and metabolites associated with response to fecal microbiota transplantation in patients with ulcerative colitis. Gastroenterology 156, 1440–1454.e2. 10.1053/j.gastro.2018.12.001 30529583

[B116] Paramsothy S. Paramsothy R. Rubin D. T. Kamm M. A. Kaakoush N. O. Mitchell H. M.. (2017). Faecal microbiota transplantation for inflammatory bowel disease: a systematic review and meta-analysis. J. Crohn's Colitis 11, 1180–1199. 10.1093/ecco-jcc/jjx063 28486648

[B117] Park H. Laffin M. R. Jovel J. Millan B. Hyun J. E. Hotte N.. (2019). The success of fecal microbial transplantation in *Clostridium difficile* infection correlates with bacteriophage relative abundance in the donor: a retrospective cohort study. Gut Microbes 10, 676–687. 10.1080/19490976.2019.1586037 30866714 PMC6867182

[B118] Parker A. Romano S. Ansorge R. Aboelnour A. Le Gall G. Savva G. M.. (2022). Fecal microbiota transfer between young and aged mice reverses hallmarks of the aging gut, eye, and brain. Microbiome 10:68. 10.1186/s40168-022-01243-w 35501923 PMC9063061

[B119] Patron R. L. Hartmann C. A. Allen S. Griesbach C. L. Kosiorek H. E. DiBaise J. K.. (2017). Vancomycin taper and risk of failure of fecal microbiota transplantation in patients with recurrent *Clostridium difficile* infection. Clin. Infect. Dis. 65, 1214–1217. 10.1093/cid/cix511 28575220

[B120] Pavanello A. Martins I. P. Tófolo L. P. Previate C. Matiusso C. Francisco F. A.. (2022). Fecal microbiota transplantation during lactation programs the metabolism of adult wistar rats in a sex-specific way. Arch. Med. Res. 53, 492–500. 10.1016/j.arcmed.2022.06.007 35840468

[B121] Peery A. F. Kelly C. R. Kao D. Vaughn B. P. Lebwohl B. Singh S.. (2024). AGA clinical practice guideline on fecal microbiota-based therapies for select gastrointestinal diseases. Gastroenterology 166, 409–434. 10.1053/j.gastro.2024.01.008 38395525

[B122] Pérez-Cobas A. E. Moya A. Gosalbes M. J. Latorre A. (2015). Colonization resistance of the gut microbiota against *Clostridium difficile*. Antibiotics 4, 337–357. 10.3390/antibiotics4030337 27025628 PMC4790290

[B123] Pinto S. Šajbenová D. Benincà E. Nooij S. Terveer E. M. Keller J. J.. (2024). Dynamics of gut microbiota after fecal microbiota transplantation in ulcerative colitis: success linked to control of prevotellaceae. J. Crohn's Colitis. 10.1093/ecco-jcc/jjae137 39225490 PMC11836888

[B124] Podlesny D. Durdevic M. Paramsothy S. Kaakoush N. O. Högenauer C. Gorkiewicz G.. (2022). Identification of clinical and ecological determinants of strain engraftment after fecal microbiota transplantation using metagenomics. Cell Rep Med. 3:100711. 10.1016/j.xcrm.2022.100711 35931074 PMC9418803

[B125] Proença I. M. Allegretti J. R. Bernardo W. M. de Moura D. Ponte Neto A. M. Matsubayashi C. O.. (2020). Fecal microbiota transplantation improves metabolic syndrome parameters: systematic review with meta-analysis based on randomized clinical trials. Nutr. Res. 83, 1–14. 10.1016/j.nutres.2020.06.018 32987284

[B126] Ramos R. J. Zhu C. Joseph D. F. Thaker S. Lacomb J. F. Markarian K.. (2022). Metagenomic and bile acid metabolomic analysis of fecal microbiota transplantation for recurrent Clostridiodes difficile and/or inflammatory bowel diseases. Med. Res. Arch. 10:10.18103/mra.v10i10.3318. 10.18103/mra.v10i10.3318 36618438 PMC9817289

[B127] Rees N. P. Shaheen W. Quince C. Tselepis C. Horniblow R. D. Sharma N.. (2022). Systematic review of donor and recipient predictive biomarkers of response to faecal microbiota transplantation in patients with ulcerative colitis. EBioMedicine 81:104088. 10.1016/j.ebiom.2022.104088 35660786 PMC9163485

[B128] Ridaura V. K. Faith J. J. Rey F. E. Cheng J. Duncan A. E. Kau A. L.. (2013). Gut microbiota from twins discordant for obesity modulate metabolism in mice. Science 341:1241214. 10.1126/science.1241214 24009397 PMC3829625

[B129] Rinott E. Youngster I. Yaskolka Meir A. Tsaban G. Zelicha H. Kaplan A.. (2021). Effects of diet-modulated autologous fecal microbiota transplantation on weight regain. Gastroenterology 160, 158–173.e10. 10.1053/j.gastro.2020.08.041 32860791 PMC7755729

[B130] Rodig N. M. Weatherly M. Kaplan A. L. Ballal S. A. Elisofon S. A. Daly K. P.. (2023). Fecal microbiota transplant in pediatric solid organ transplant recipients. Transplantation 107, 2073–2077. 10.1097/TP.0000000000004656 37211643

[B131] Rossen N. G. Fuentes S. van der Spek M. J. Tijssen J. G. Hartman J. H. Duflou A.. (2015). Findings from a randomized controlled trial of fecal transplantation for patients with ulcerative colitis. Gastroenterology 149, 110–118.e4. 10.1053/j.gastro.2015.03.045 25836986

[B132] Ruan W. Engevik M. A. Spinler J. K. Versalovic J. (2020). Healthy human gastrointestinal microbiome: composition and function after a decade of exploration. Dig. Dis. Sci. 65, 695–705. 10.1007/s10620-020-06118-4 32067143

[B133] Schmidt T. Li S. S. Maistrenko O. M. Akanni W. Coelho L. P. Dolai S.. (2022). Drivers and determinants of strain dynamics following fecal microbiota transplantation. Nat. Med. 28, 1902–1912. 10.1038/s41591-022-01913-0 36109636 PMC9499871

[B134] Seekatz A. M. Aas J. Gessert C. E. Rubin T. A. Saman D. M. Bakken J. S.. (2014). Recovery of the gut microbiome following fecal microbiota transplantation. MBio 5, e00893–e00814. 10.1128/mBio.00893-14 24939885 PMC4068257

[B135] Sehgal K. Yadav D. Saha S. Mara K. Grover M. Khanna S. (2024). Sex-Discordant Fecal microbiota transplantation for *Clostridioides difficile* may increase risk of postinfection irritable bowel syndrome. Gastroenterology 166, 704–706.e2. 10.1053/j.gastro.2023.11.295 38056511

[B136] Serrano-Villar S. Talavera-Rodríguez A. Gosalbes M. J. Madrid N. Pérez-Molina J. A. Elliott R. J.. (2021). Fecal microbiota transplantation in HIV: a pilot placebo-controlled study. Nat. Commun. 12:1139. 10.1038/s41467-021-21472-1 33602945 PMC7892558

[B137] Settanni C. R. Ianiro G. Bibb,ò S. Cammarota G. Gasbarrini A. (2021). Gut microbiota alteration and modulation in psychiatric disorders: current evidence on fecal microbiota transplantation. Prog. Neuropsychopharmacol. Biol. Psychiatry 109:110258. 10.1016/j.pnpbp.2021.110258 33497754

[B138] Sheng L. Jena P. K. Hu Y. Wan Y. Y. (2021). Age-specific microbiota in altering host inflammatory and metabolic signaling as well as metabolome based on the sex. Hepatobiliary Surg. Nutr. 10, 31–48. 10.21037/hbsn-20-671 33575288 PMC7867716

[B139] Shogbesan O. Poudel D. R. Victor S. Jehangir A. Fadahunsi O. Shogbesan G.. (2018). A systematic review of the efficacy and safety of fecal microbiota transplant for *Clostridium difficile* infection in immunocompromised patients. Can J Gastroenterol Hepatol 2018, 1394379. 10.1155/2018/1394379 30246002 PMC6139215

[B140] Shtossel O. Turjeman S. Riumin A. Goldberg M. R. Elizur A. Bekor Y.. (2023). Recipient-independent, high-accuracy FMT-response prediction and optimization in mice and humans. Microbiome 11:181. 10.1186/s40168-023-01623-w 37580821 PMC10424414

[B141] Shukla R. Ghoshal U. Dhole T. N. Ghoshal U. C. (2015). Fecal microbiota in patients with irritable bowel syndrome compared with healthy controls using real-time polymerase chain reaction: an evidence of dysbiosis. Dig. Dis. Sci. 60, 2953–2962. 10.1007/s10620-015-3607-y 25784074

[B142] Singh P. Alm E. J. Kelley J. M. Cheng V. Smith M. Kassam Z.. (2022). Effect of antibiotic pretreatment on bacterial engraftment after Fecal Microbiota Transplant (FMT) in IBS-D. Gut Microbes 14:2020067. 10.1080/19490976.2021.2020067 35014601 PMC8757476

[B143] Smillie C. S. Sauk J. Gevers D. Friedman J. Sung J. Youngster I.. (2018). Strain tracking reveals the determinants of bacterial engraftment in the human gut following fecal microbiota transplantation. Cell Host Microbe 23, 229–240.e5. 10.1016/j.chom.2018.01.003 29447696 PMC8318347

[B144] Soldi S. Vasileiadis S. Lohner S. Uggeri F. Puglisi E. Molinari P.. (2019). Prebiotic supplementation over a cold season and during antibiotic treatment specifically modulates the gut microbiota composition of 3-6 year-old children. Benef. Microbes 10, 253–263. 10.3920/BM2018.0116 30776899

[B145] Song Y. N. Yang D. Y. Veldhuyzen van Zanten S. Wong K. McArthur E. Song C. Z.. (2022). Fecal microbiota transplantation for severe or fulminant *clostridioides difficile* infection: systematic review and meta-analysis. J. Can. Assoc. Gastroenterol. 5, e1–e11. 10.1093/jcag/gwab023 35118227 PMC8806043

[B146] Sood A. Singh A. Mahajan R. Midha V. Kaur K. Singh D.. (2020). Clinical Predictors of response to faecal microbiota transplantation in patients with active ulcerative colitis. J. Crohn's Colitis. 10.1093/ecco-jcc/jjaa163 32772093

[B147] Spindelboeck W. Schulz E. Uhl B. Kashofer K. Aigelsreiter A. Zinke-Cerwenka W.. (2017). Repeated fecal microbiota transplantations attenuate diarrhea and lead to sustained changes in the fecal microbiota in acute, refractory gastrointestinal graft-versus-host-disease. Haematologica 102, e210–e213. 10.3324/haematol.2016.154351 28154090 PMC5477627

[B148] Staley C. Kaiser T. Vaughn B. P. Graiziger C. T. Hamilton M. J. Rehman T. U.. (2018). Predicting recurrence of *Clostridium difficile* infection following encapsulated fecal microbiota transplantation. Microbiome 6, 166. 10.1186/s40168-018-0549-6 30227892 PMC6145197

[B149] Stripling J. Kumar R. Baddley J. W. Nellore A. Dixon P. Howard D.. (2015). Loss of Vancomycin-resistant enterococcus fecal dominance in an organ transplant patient with *Clostridium difficile* colitis after fecal microbiota transplant. Open Forum Infect Dis 2, ofv078. 10.1093/ofid/ofv078 26180828 PMC4498259

[B150] Sulaiman J. E. Thompson J. Qian Y. Vivas E. I. Diener C. Gibbons S. M.. (2024). Elucidating human gut microbiota interactions that robustly inhibit diverse *Clostridioides difficile* strains across different nutrient landscapes. Nat. Commun. 15:7416. 10.1038/s41467-024-51062-w 39198411 PMC11358386

[B151] Tanaka T. Talegawkar S. A. Jin Y. Candia J. Tian Q. Moaddel R.. (2022). Metabolomic profile of different dietary patterns and their association with frailty index in community-dwelling older men and women. Nutrients 14:2237. 10.3390/nu14112237 35684039 PMC9182888

[B152] Tang L. L. Feng W. Z. Cheng J. J. Gong Y. N. (2020). Clinical remission of ulcerative colitis after different modes of faecal microbiota transplantation: a meta-analysis. Int. J. Colorectal Dis. 35, 1025–1034. 10.1007/s00384-020-03599-7 32388604

[B153] Tunali V. Arslan N. Ç. Ermiş B. H. Derviş Hakim G. Gündogdu A. Hora M.. (2024). A multicenter randomized controlled trial of microbiome-based artificial intelligence-assisted personalized diet vs low-fermentable oligosaccharides, disaccharides, monosaccharides, and polyols diet: a novel approach for the management of irritable bowel syndrome. Am. J. Gastroenterol. 119, 1901–1912. 10.14309/ajg.0000000000002862 38717025 PMC11365594

[B154] Urtecho G. Moody T. Huang Y. Sheth R. U. Richardson M. Descamps H. C.. (2024). Spatiotemporal dynamics during niche remodeling by super-colonizing microbiota in the mammalian gut. Cell Syst 15, 1002–1017.e4. 10.1016/j.cels.2024.10.007 39541983 PMC12066173

[B155] Vendrik K. Terveer E. M. Kuijper E. J. Nooij S. Boeije-Koppenol E. Sanders I.. (2021). Periodic screening of donor faeces with a quarantine period to prevent transmission of multidrug-resistant organisms during faecal microbiota transplantation: a retrospective cohort study. Lancet Infect. Dis. 21, 711–721. 10.1016/S1473-3099(20)30473-4 33275940

[B156] Vich Vila A. Zhang J. Liu M. Faber K. N. Weersma R. K. (2024). Untargeted faecal metabolomics for the discovery of biomarkers and treatment targets for inflammatory bowel diseases. Gut 73, 1909–1920. 10.1136/gutjnl-2023-329969 39002973 PMC11503092

[B157] Wang J. Zhong Y. Zhu H. Mahgoub O. K. Jian Z. Gu L.. (2022). Different gender-derived gut microbiota influence stroke outcomes by mitigating inflammation. J. Neuroinflammat. 19, 245. 10.1186/s12974-022-02606-8 36195899 PMC9531521

[B158] Wang J. J. Wang J. Pang X. Y. Zhao L. P. Tian L. Wang X. P. (2016). Sex differences in colonization of gut microbiota from a man with short-term vegetarian and inulin-supplemented diet in germ-free mice. Sci. Rep. 6:36137. 10.1038/srep36137 27796317 PMC5086848

[B159] Wang T. Shi Z. Ren H. Xu M. Lu J. Yang F.. (2024). Divergent age-associated and metabolism-associated gut microbiome signatures modulate cardiovascular disease risk. Nat. Med. 30, 1722–1731. 10.1038/s41591-024-03038-y 38844795

[B160] Wang Y. Wiesnoski D. H. Helmink B. A. Gopalakrishnan V. Choi K. DuPont H. L.. (2018). Fecal microbiota transplantation for refractory immune checkpoint inhibitor-associated colitis. Nat. Med. 24, 1804–1808. 10.1038/s41591-018-0238-9 30420754 PMC6322556

[B161] Wang Y. Zhang S. Borody T. J. Zhang F. (2022). Encyclopedia of fecal microbiota transplantation: a review of effectiveness in the treatment of 85 diseases. Chin. Med. J. 135, 1927–1939. 10.1097/CM9.0000000000002339 36103991 PMC9746749

[B162] Watane A. Cavuoto K. M. Rojas M. Dermer H. Day J. O. Banerjee S.. (2022). Fecal microbial transplant in individuals with immune-mediated dry eye. Am. J. Ophthalmol. 233, 90–100. 10.1016/j.ajo.2021.06.022 34214453 PMC8678170

[B163] Wei S. Bahl M. I. Baunwall S. Dahlerup J. F. Hvas C. L. Licht T. R. (2022). Gut microbiota differs between treatment outcomes early after fecal microbiota transplantation against recurrent *Clostridioides difficile* infection. Gut Microbes 14:2084306. 10.1080/19490976.2022.2084306 36519447 PMC9176232

[B164] Wei Y. Gong J. Zhu W. Tian H. Ding C. Gu L.. (2016). Pectin enhances the effect of fecal microbiota transplantation in ulcerative colitis by delaying the loss of diversity of gut flora. BMC Microbiol. 16:255. 10.1186/s12866-016-0869-2 27809778 PMC5095982

[B165] Weingarden A. R. Chen C. Bobr A. Yao D. Lu Y. Nelson V. M.. (2014). Microbiota transplantation restores normal fecal bile acid composition in recurrent *Clostridium difficile* infection. Am. J. Physiol. Gastrointest. Liver Physiol. 306, G310–319. 10.1152/ajpgi.00282.2013 24284963 PMC3920123

[B166] Wilson B. C. Vatanen T. Cutfield W. S. O'Sullivan J. M. (2019). The super-donor phenomenon in fecal microbiota transplantation. Front. Cell. Infect. Microbiol. 9:2. 10.3389/fcimb.2019.00002 30719428 PMC6348388

[B167] Wilson B. C. Vatanen T. Jayasinghe T. N. Leong K. Derraik J. Albert B. B.. (2021). Strain engraftment competition and functional augmentation in a multi-donor fecal microbiota transplantation trial for obesity. Microbiome 9:107. 10.1186/s40168-021-01060-7 33985595 PMC8120839

[B168] Witjes J. J. Smits L. P. Pekmez C. T. Prodan A. Meijnikman A. S. Troelstra M. A.. (2020). Donor fecal microbiota transplantation alters gut microbiota and metabolites in obese individuals with Steatohepatitis. Hepatol Commun 4, 1578–1590. 10.1002/hep4.1601 33163830 PMC7603524

[B169] Woodworth M. H. Conrad R. E. Haldopoulos M. Pouch S. M. Babiker A. Mehta A. K.. (2023). Fecal microbiota transplantation promotes reduction of antimicrobial resistance by strain replacement. Sci. Transl. Med. 15:eabo2750. 10.1126/scitranslmed.abo2750 37910603 PMC10821315

[B170] Wu Q. Boonma P. Badu S. Yalcinkaya N. So S. Y. Garey K. W.. (2023). Donor-recipient specificity and age-dependency in fecal microbiota therapy and probiotic resolution of gastrointestinal symptoms. NPJ Biofilms Microbiom. 9:54. 10.1038/s41522-023-00421-4 37537181 PMC10400536

[B171] Wu X. Li P. Wang W. Xu J. Ai R. Wen Q.. (2023). The underlying changes in serum metabolic profiles and efficacy prediction in patients with extensive ulcerative colitis undergoing fecal microbiota transplantation. Nutrients 15:3340. 10.3390/nu15153340 37571277 PMC10421017

[B172] Xiao Y. Angulo M. T. Lao S. Weiss S. T. Liu Y. Y. (2020). An ecological framework to understand the efficacy of fecal microbiota transplantation. Nat. Commun. 11:3329. 10.1038/s41467-020-17180-x 32620839 PMC7334230

[B173] Yakout A. Bi Y. Harris D. M. (2024). *Clostridioides difficile*: a concise review of best practices and updates. J. Prim. Care Community Health 15:21501319241249645. 10.1177/21501319241249645 38726585 PMC11085020

[B174] Yang H. Wu X. Li X. Zang W. Zhou Z. Zhou Y.. (2024). A commensal protozoan attenuates *Clostridioides difficile* pathogenesis in mice via arginine-ornithine metabolism and host intestinal immune response. Nat. Commun. 15:2842. 10.1038/s41467-024-47075-0 38565558 PMC10987486

[B175] Yang Y. Zheng X. Wang Y. Tan X. Zou H. Feng S.. (2022). Human fecal microbiota transplantation reduces the susceptibility to dextran sulfate sodium-induced germ-free mouse colitis. Front. Immunol. 13:836542. 10.3389/fimmu.2022.836542 35237276 PMC8882623

[B176] Yau Y. K. Su Q. Xu Z. Tang W. Ching J. Mak J.. (2023). Randomised clinical trial: Faecal microbiota transplantation for irritable bowel syndrome with diarrhoea. Aliment. Pharmacol. Ther. 58, 795–804. 10.1111/apt.17703 37667968

[B177] Yurkovetskiy L. Burrows M. Khan A. A. Graham L. Volchkov P. Becker L.. (2013). Gender bias in autoimmunity is influenced by microbiota. Immunity 39, 400–412. 10.1016/j.immuni.2013.08.013 23973225 PMC3822899

[B178] Zellmer C. Sater M. Huntley M. H. Osman M. Olesen S. W. Ramakrishna B. (2021). Shiga toxin-producing escherichia coli transmission via fecal microbiota transplant. Clin. Infect. Dis. 72, e876–e880. 10.1093/cid/ciaa1486 33159210

[B179] Zhang F. Zuo T. Yeoh Y. K. Cheng F. Liu Q. Tang W.. (2021). Longitudinal dynamics of gut bacteriome, mycobiome and virome after fecal microbiota transplantation in graft-versus-host disease. Nat. Commun. 12:65. 10.1038/s41467-020-20240-x 33397897 PMC7782528

[B180] Zhang S. Lu G. Wang W. Li Q. Wang R. Zhang Z.. (2024). A predictive machine-learning model for clinical decision-making in washed microbiota transplantation on ulcerative colitis. Comput. Struct. Biotechnol. J. 24, 583–592. 10.1016/j.csbj.2024.08.021 39281978 PMC11399476

[B181] Zhang X. Luo X. Tian L. Yue P. Li M. Liu K.. (2023). The gut microbiome dysbiosis and regulation by fecal microbiota transplantation: umbrella review. Front. Microbiol. 14:1286429. 10.3389/fmicb.2023.1286429 38029189 PMC10655098

[B182] Zhang Y. Liu Q. Yu Y. Wang M. Wen C. He Z. (2020). Early and short-term interventions in the gut microbiota affects lupus severity, progression, and treatment in MRL/lpr mice. Front. Microbiol. 11:628. 10.3389/fmicb.2020.00628 32346376 PMC7171286

[B183] Zhang Y. Wang S. Wang H. Cao M. Wang M. Zhang B.. (2024). Efficacy of donor-recipient-matched faecal microbiota transplantation in patients with IBS-D: a single-centre, randomized, double-blind placebo-controlled study. Digestion 105, 457–467. 10.1159/000540420 39084197

[B184] Zhang Y. Zhu H. Du S. Wang H. Li H. Wang M.. (2023). Medium-chain and long-chain fatty acids are associated with diarrheal predominant irritable bowel syndrome revealed by DESI-MSI. J. Gastroenterol. 58, 1124–1133. 10.1007/s00535-023-02030-6 37578536 PMC10590296

[B185] Zhang Z. Mocanu V. Deehan E. C. Hotte N. Zhu Y. Wei S.. (2024). Recipient microbiome-related features predicting metabolic improvement following fecal microbiota transplantation in adults with severe obesity and metabolic syndrome: a secondary analysis of a phase 2 clinical trial. Gut Microbes 16:2345134. 10.1080/19490976.2024.2345134 38685731 PMC11062372

[B186] Zhong Y. Cao J. Deng Z. Ma Y. Liu J. Wang H. (2021). Effect of fiber and fecal microbiota transplantation donor on recipient mice gut microbiota. Front. Microbiol. 12:757372. 10.3389/fmicb.2021.757372 34721365 PMC8548821

[B187] Zhu J. Yin J. Chen J. Hu M. Lu W. Wang H.. (2024). Integrative analysis with microbial modelling and machine learning uncovers potential alleviators for ulcerative colitis. Gut Microbes 16:2336877. 10.1080/19490976.2024.2336877 38563656 PMC10989691

[B188] Zhu X. Huang X. Hu M. Sun R. Li J. Wang H.. (2024). A specific enterotype derived from gut microbiome of older individuals enables favorable responses to immune checkpoint blockade therapy. Cell Host Microbe 32, 489–505.e5. 10.1016/j.chom.2024.03.002 38513657

[B189] Zoll J. Read M. N. Heywood S. E. Estevez E. Marshall J. Kammoun H. L.. (2020). Fecal microbiota transplantation from high caloric-fed donors alters glucose metabolism in recipient mice, independently of adiposity or exercise status. Am. J. Physiol. Endocrinol. Metab. 319, E203–E216. 10.1152/ajpendo.00037.2020 32516027

[B190] Zou M. Jie Z. Cui B. Wang H. Feng Q. Zou Y.. (2020). Fecal microbiota transplantation results in bacterial strain displacement in patients with inflammatory bowel diseases. FEBS Open Bio. 10, 41–55. 10.1002/2211-5463.12744 31622538 PMC6943227

[B191] Zuo T. Wong S. H. Cheung C. P. Lam K. Lui R. Cheung K.. (2018b). Gut fungal dysbiosis correlates with reduced efficacy of fecal microbiota transplantation in *Clostridium difficile* infection. Nat. Commun. 9:3663. 10.1038/s41467-018-06103-6 30202057 PMC6131390

[B192] Zuo T. Wong S. H. Lam K. Lui R. Cheung K. Tang W.. (2018a). Bacteriophage transfer during faecal microbiota transplantation in *Clostridium difficile* infection is associated with treatment outcome. Gut 67, 634–643. 10.1136/gutjnl-2017-313952 28539351 PMC5868238

